# Transition From Targeted Breeding to Mainstreaming of Biofortification Traits in Crop Improvement Programs

**DOI:** 10.3389/fpls.2021.703990

**Published:** 2021-09-14

**Authors:** Parminder S. Virk, Meike S. Andersson, Jairo Arcos, Mahalingam Govindaraj, Wolfgang H. Pfeiffer

**Affiliations:** ^1^HarvestPlus, International Food Policy Research Institute (IFPRI), Washington, DC, United States; ^2^Alliance of Bioversity International and the International Center for Tropical Agriculture (CIAT), Cali, Colombia; ^3^Crop Improvement, International Crops Research Institute for the Semi-Arid Tropics (ICRISAT), Patancheru, India

**Keywords:** iron, zinc, mainstreaming, biofortification approach, provitamin A

## Abstract

Biofortification breeding for three important micronutrients for human health, namely, iron (Fe), zinc (Zn), and provitamin A (PVA), has gained momentum in recent years. HarvestPlus, along with its global consortium partners, enhances Fe, Zn, and PVA in staple crops. The strategic and applied research by HarvestPlus is driven by product-based impact pathway that integrates crop breeding, nutrition research, impact assessment, advocacy, and communication to implement country-specific crop delivery plans. Targeted breeding has resulted in 393 biofortified crop varieties by the end of 2020, which have been released or are in testing in 63 countries, potentially benefitting more than 48 million people. Nevertheless, to reach more than a billion people by 2030, future breeding lines that are being distributed by Consultative Group on International Agricultural Research (CGIAR) centers and submitted by National Agricultural Research System (NARS) to varietal release committees should be biofortified. It is envisaged that the mainstreaming of biofortification traits will be driven by high-throughput micronutrient phenotyping, genomic selection coupled with speed breeding for accelerating genetic gains. It is noteworthy that targeted breeding gradually leads to mainstreaming, as the latter capitalizes on the progress made in the former. Efficacy studies have revealed the nutritional significance of Fe, Zn, and PVA biofortified varieties over non-biofortified ones. Mainstreaming will ensure the integration of biofortified traits into competitive varieties and hybrids developed by private and public sectors. The mainstreaming strategy has just been initiated in select CGIAR centers, namely, International Maize and Wheat Improvement Center (CIMMYT), International Rice Research Institute (IRRI), International Crops Research Institute for the Semi-Arid Tropics (ICRISAT), International Institute of Tropical Agriculture (IITA), and International Center for Tropical Agriculture (CIAT). This review will present the key successes of targeted breeding and its relevance to the mainstreaming approaches to achieve scaling of biofortification to billions sustainably.

## Introduction

Globally, micronutrient malnutrition or hidden hunger is affecting more than 2 billion people according to the 2014 Global Hunger Index [(IFPRI (International Food Policy Research Institute), 2014); (FAO, 2019)]. Malnutrition is primarily a result of one or more micronutrient deficiencies and is recognized as a serious human health problem in the twenty first century. Among the most striking of these are vitamin A, iron (Fe), iodine (I), and zinc (Zn) deficiencies (Bailey et al., 2015). Historical interventions, such as pharmaceutical supplementation, industrial food fortification, and dietary diversification, have long been used to address this problem. The impact of food fortification is well-known, which has been successful primarily for vitamin A and iodized salt, and supplementation has been hugely successful in addressing vitamin A deficiency. Dietary diversification will continue to have relevance; however, it is subject to affordability. Biofortification endeavors to serve hard to reach rural communities as well as peri-urban and urban consumers (Stein et al., 2005).

Biofortification refers to a breeding process by which the essential mineral and vitamin concentration of staple food crops are increased through conventional plant breeding and/or genetic modification, and the application of micronutrient fertilizers to crops. Genetic biofortification (heritable) has proven to be cost-effective and sustainable, as it takes the advantage to ensure nutritional value from one crop generation to another and serves the rural poor [(CAST (Council for Agricultural Science and Technology), 2020)]. Nevertheless, the potential of genetic biofortification benefits from recommended agronomic practices and complements agronomic biofortification. Furthermore, as biofortified varieties are grown and consumed, their adoption does not require a change in ongoing dietary habits. Nonetheless, deficiencies of these nutrients and vitamins cannot be addressed by a single food crop or by a single intervention. Hence, biofortification is likely to complement existing nutritional strategies. Biofortified crops are being developed by the Consultative Group on International Agricultural Research (CGIAR) HarvestPlus program through an interdisciplinary alliance of research institutions and implementing partners, in particular, of primary staples such as rice (for Zn), wheat (for Zn), maize (for Zn and PVA), cassava (for PVA), sweet potato (for PVA), beans (for iron), and pearl millet (for iron), based on crop and nutrient combination, hereafter referred to as zinc rice, zinc wheat, zinc maize, iron beans, iron pearl millet, provitamin A (PVA) maize, PVA cassava, and PVA sweet potato, which have been published in many studies [Andersson et al., 2017; Bouis, 2018; CAST (Council for Agricultural Science and Technology), 2020]. In principle, biofortification target increments of micronutrients are expected to be met without compromising yield productivity or agronomic performance in the target crops. This is evidenced by partnership-based biofortification breeding, testing, and scaling-up. For instance, about 10 million farming households are growing, while > 48 million farming household level people are potentially benefiting from biofortified crops in > 30 countries [(CAST (Council for Agricultural Science and Technology), 2020)].

The impact of coronavirus disease-2019 (COVID-19) is likely to adversely impact household incomes, especially in low and middle-income countries (LMICs) that work in farming and rural regions of developing countries. The situation will impair the food purchasing power of vulnerable populations. Reduced household incomes often translate to no or little affordability to animal- and fruit-/vegetable-based foods, increasing reliance on locally grown staple foods to meet caloric needs (Bouis et al., 2011). Therefore, biofortification needs to be accelerated to disseminate more nutrient-dense varieties to the vulnerable. During the COVID-19 pandemic, disrupted or restricted mobility of farm produce jeopardized food supply chain in Asian and African countries, that would lead to increase in food prices post-COVID-19. This is likely to make food more expensive and potentially out of the reach of poor families (Heck et al., 2020). Hence, poor income, increasing prices of nutrient-dense foods, and shifting policy priorities caused by COVID-19 are likely to increase the demand for biofortified staples as a local and affordable source of micronutrients. The impact of the current pandemic may be extended to cultivar testing and release, and seed production, disrupting supply chains.

This review aims to summarize the current targeted breeding strategy for the development of biofortified primary staple crops and to inform the prospects of mainstreaming breeding strategies to maximize genetic gains for nutritional and agronomic traits.

## Targeted Breeding Approach

Targeted breeding for micronutrient contents was conceived by HarvestPlus, a challenge program of the CGIAR. This targeted breeding (TB) approach is largely focused on staple crop-trait prioritization for target countries and was based on Biofortification Priority Index (BPI), which basically ranks low- and middle-income countries according to the prevalence of malnutrition (https://bpi.harvestplus.org). Establishing global biofortification coordination to demonstrate nutritional enhancement through crop breeding is highly feasible in a wide range of food crops (cereals, legumes, tubers) and is a major part of TB. In fact, TB involves breeding for a given production zone usually defined by abiotic and biotic environmental factors, growing conditions, and consumer preferences. Briefly, TB aims to continuously improve the germplasm and early-stage breeding pipeline of crops for given nutrients through building a breeding capacity with CGIAR and partner breeding centers. Target zones for the public (National Agricultural Research Systems-NARS) and private sectors are, in general, within a given country. The breeding programs of CG centers, in general, are focused on broader geographic regions i.e., many target zones of a crop. These target zones are so-called “Mega-Environments” (MEs) or Target Population of Environments (TPEs) and are frequently transcontinental. TB comprises several stages and is described briefly hereunder.

### Setting the Breeding Targets

During the conceptual development of biofortification targeted breeding, a global working group, which consisted of nutritionists and plant breeders, established nutritional breeding target increments based on the quantity of various staple foods consumption in a given agro-ecological zone, considering nutrient losses during storage and processing, and nutrient bioavailability in respective crops at that time. Breeding increment targets for biofortified crops were designed to meet the nutritional requirements in a population, particularly for women and children as they are the most vulnerable groups in developing countries. Breeding targets for primary staple crops were first proposed by Hotz and McClafferty (2007) and were later revised as more data became available and accessible [Andersson et al., 2017; Bouis and Saltzman, 2017; CAST (Council for Agricultural Science and Technology), 2020]. The baseline of each essential micronutrient and vitamin was evaluated for each crop and reported in wheat (25 ppm Zn), rice (16 ppm Zn), maize (25 ppm Zn), beans (50 ppm Fe), pearl millet (47 ppm Fe), maize (0 ppm PVA), sweet potato (2 ppm PVA), and cassava (0 ppm PVA) [Beebe et al., 2000; Bouis and Saltzman, 2017; Govindaraj et al., 2019; CAST (Council for Agricultural Science and Technology), 2020]. Most of the baseline is based on cultivars commercially grown by farmers and pipelines. TB targets are measured from this crop-specific baseline. For Zn crops, namely, wheat, rice, and maize, the target increment was set at +12 ppm; and for iron crops, it was set at +30 and +44 ppm for pearl millet and beans, respectively. For cassava and maize, the provitamin A target was set at +15 ppm, while it was +70 ppm for sweet potato [Bouis and Saltzman, 2017; CAST (Council for Agricultural Science and Technology), 2020].

### High-Throughput Phenotyping for Micronutrient Densities

The development and availability of cost-effective and high-throughput analytical methods for the unambiguous micronutrient estimation in grain and tuber crops were also identified as a prerequisite for TB. A large number of samples per season were screened throughout the TB process. The key phenotyping methods, along with the adoption and modification of equipment, are described in detail by Pfeiffer and McClafferty (2007), Andersson et al. (2017), and CAST (Council for Agricultural Science and Technology) (2020). A detailed review and the current status of high-throughput screening methodologies are presented by Guild et al. (2017). In general, destructive chemical digestion-based wet lab instruments, such as those used for atomic absorption spectroscopy (AAS) or inductively coupled plasma optical emission spectrometry (ICP-OES), have been used to assess the estimation of plant minerals. Considering crop cycle time and rapid turnaround, the non-destructive method is faster and convenient to screen thousands of breeding and germplasm samples in a given time. Hence, x-ray fluorescence spectroscopy (XRF) and near-infrared spectroscopy (NIRS) have emerged as the methods of choice, as they require minimal pre-analysis preparation and lower costs (Paltridge et al., 2012a,b). The sample output of XRF is much higher than ICP; for instance, about 250–300 samples can be processed per day (Govindaraj et al., 2019). Therefore, XRF- and NIRS-based high throughput cost-effective phenotypic tools for mineral and carotenoid measurements are the current choice for breeding programs.

### Prioritization of Crop-Nutrient Combinations and Breeding Centers

The following criteria were used to identify crop-nutrient combinations and CG centers dependent on crop expertise.

1. Availability of germplasm for high-nutrient parents with nutrient target levels.
2. Additional increments of Fe, Zn, and PVA contents are required to have a measurable public health impact.
3. Keeping in mind to maximize impact (number of potential malnourished consumers with high per capita consumption, breeding capacity of centers), crop-nutrient and center combinations were identified (Table 1).

**Table 1:** Prioritization of crop-nutrient combinations and Consultative Group on International Agricultural Research (CGIAR) centers involved in targeted breeding.

**Crop**	**Nutrient**	**Center(s)**
Sweetpotato	Provitamin A (PVA)	CIP
PearlMillet	Iron (Fe)	ICRISAT
Beans	Iron (Fe)	CIAT
Cassava	Provitamin A (PVA)	CIAT,IITA
Maize	Provitamin A (PVA)	CIMMYT,IITA
Maize	Zinc (Zn)	CIMMYT,IITA
Wheat	Zinc (Zn)	CIMMYT
Rice	Zinc (Zn)	IRRI, CIAT

### Prioritization of Countries-Crops Combinations

Countries and crops were identified to maximize the impact of biofortification in TB phase. Two criteria were used in this exercise: (1) the extent of micronutrient deficiencies in target country populations and (2) the greatest potential impact of targeted biofortified crops. A combination of different indices was employed by various experts (Bouis and Saltzman, 2017) to identify the following countries-crops combinations (Table 2). However, in the mainstreaming phase, these criteria may be revisited. For more details, please refer to https://www.harvestplus.org/knowledge-market/in-the-news/scaling-biofortified-crops-which-ones-where-and-when.

**Table 2:** Priority countries-primary crops combination.

**Country**	**Biofortified Crops**
Bangladesh	Sweet potato, Rice
Benin	Pearl millet
Brazil	Maize, Cassava, Sweet potato, Beans
China	Sweet potato, Wheat, Rice
Colombia	Beans
DR Congo	Maize, Cassava, Beans
Egypt	Sweet potato
Ethiopia	Sweet potato
Ghana	Maize, Cassava, Sweet potato
Guatemala	Beans
India	Sweet potato, wheat, Rice, Pearl millet
Indonesia	Sweet potato
Kenya	Sweet potato, Beans
Madagascar	Sweet potato
Malawi	Maize, Cassava, Sweet potato, Beans
Mali	Maize
Mozambique	Sweet potato
Niger	Pearl millet
Nigeria	Maize, Cassava, Sweet potato
Pakistan	Wheat
Rwanda	Maize, Sweet potato, Beans
Tanzania	Sweet potato
Uganda	Sweet potato, Beans
Vietnam	Sweet potato
Zambia	Maize, Sweet potato
Zimbabwe	Maize, Beans

### Traits Efficacy

Over 48 million people worldwide are potentially consuming biofortified crops [Bouis and Saltzman, 2017; CAST (Council for Agricultural Science and Technology), 2020]. It has now been recognized that biofortified crops have the potential to provide an additional 20 to 100% of the Estimated Average Requirement (EAR; median daily intake value estimated to meet the requirement of half the healthy individuals in a life-stage and gender group) for specific nutrients based on per capita consumption (https://www.harvestplus.org/content/estimated-average-requirements-provided-biofortification). The availability of screening methodologies and the setting of breeding targets for micronutrients and other supporting materials pave the way for starting biofortification [Govindaraj et al., 2016, 2019; CAST (Council for Agricultural Science and Technology), 2020].

**Figure 1:**
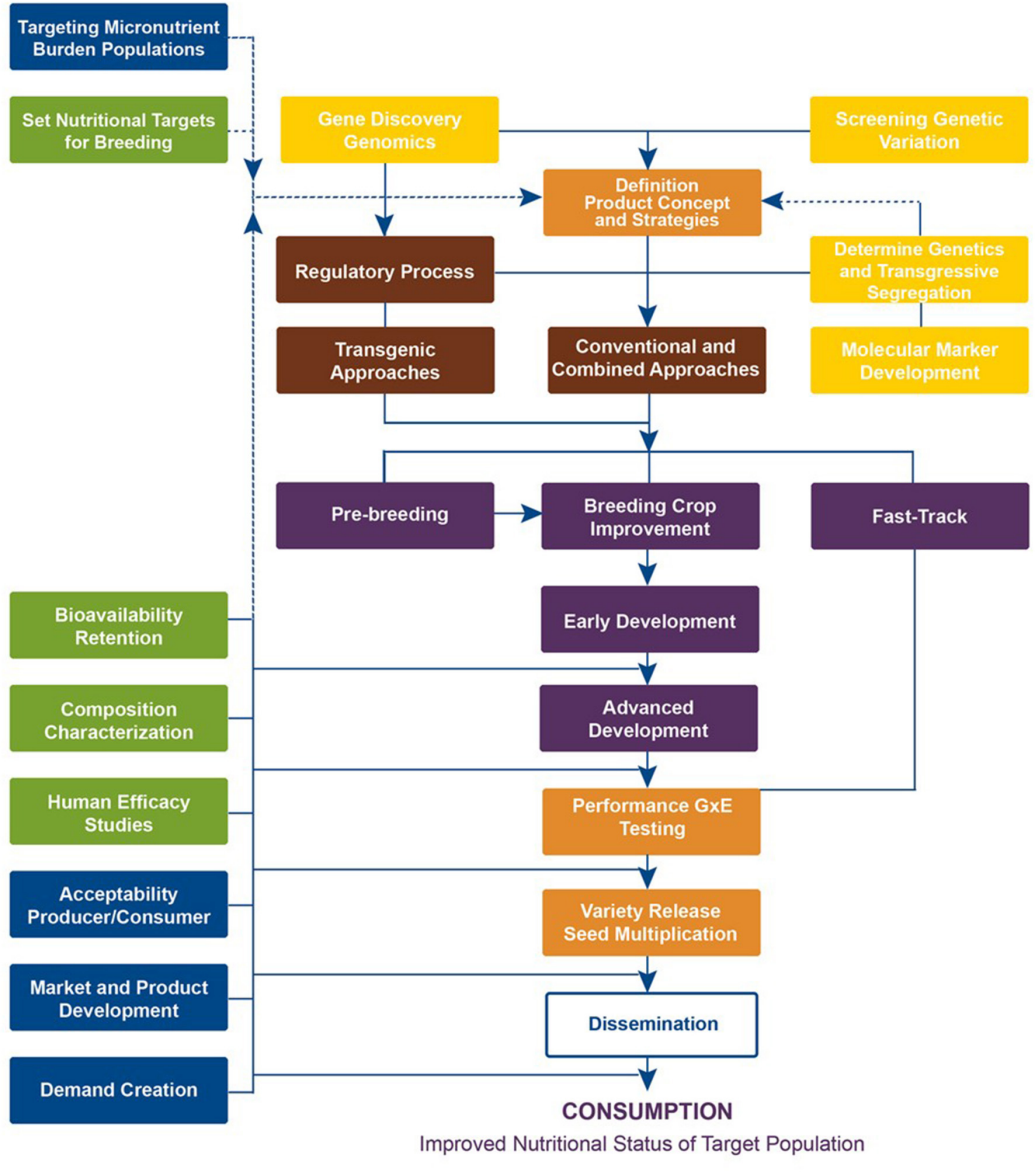
Crop Development Conceptual Framework followed by HarvestPlus (source: www.harvestplus.org).

Targeted breeding requires the availability of sufficient genetic variation in the germplasm for the target micronutrient, as highlighted by yellow boxes in the crop development conceptual framework (Figure 1). Plant breeders screen existing active germplasm collections, including released varieties, advanced germplasm, and accessions in international and national gene banks. This serves two purposes: (1) identify already adapted germplasm with previously unknown micronutrient traits for “fast-tracking” in which these are either released or relaunched as a biofortified variety and (2) identify donor parents to be used in making new crosses, developing molecular markers, related genetic studies, etc. If the donor parents are identified from unadapted landraces, pre-breeding is necessary before using them in breeding programs. However, if adequate crop genetic variation is present in the adapted germplasm and parents, then selected donor parents can be directly utilized in the breeding program (purple boxes). Fortunately, a wide range of vitamin and mineral concentrations was available to start biofortification (reviewed by Andersson et al., 2017). The promising parental germplasm was used in the early stage of product development and further parent building to set up breeding pipelines to advance to the next stage. This process largely takes place in the breeding center.

**Figure 2:**
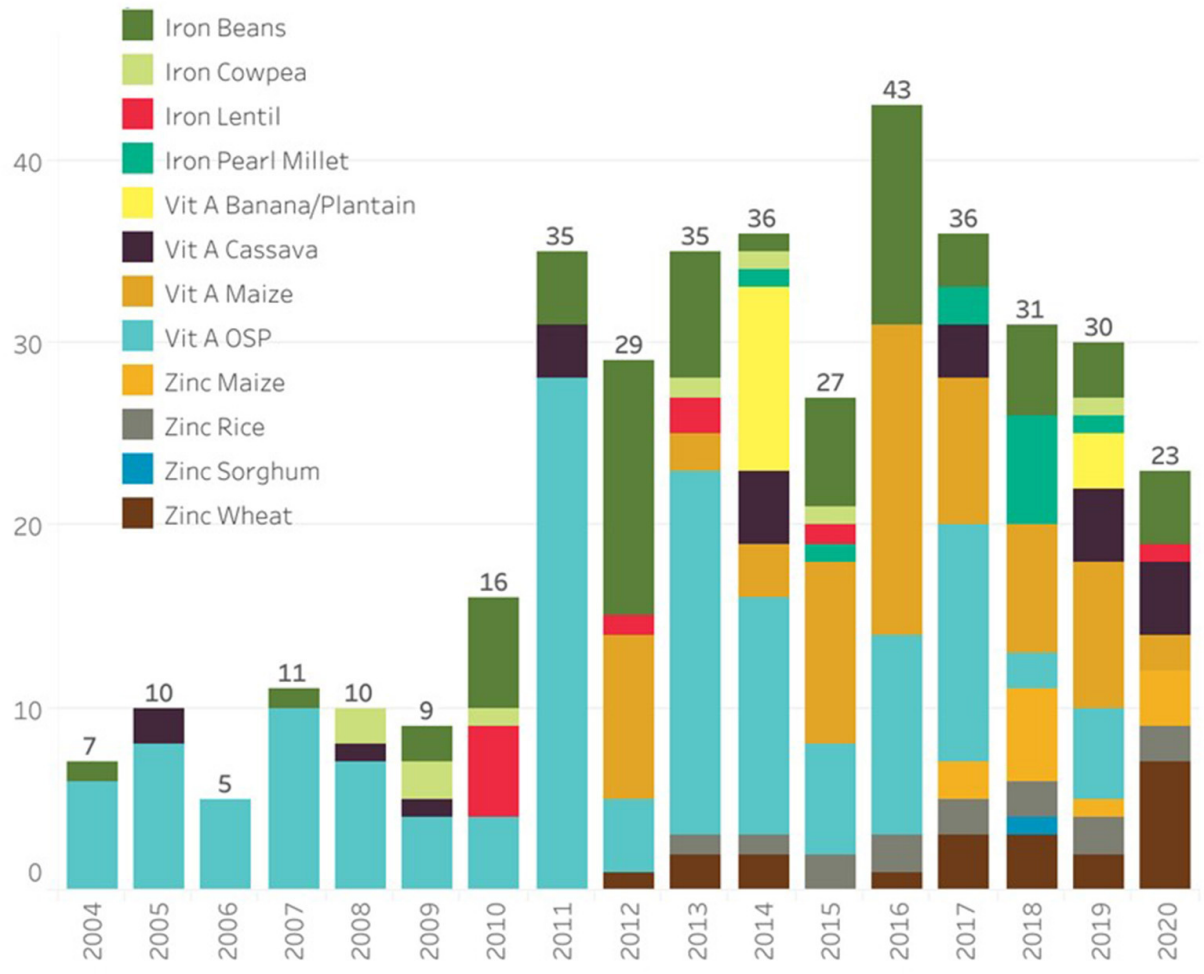
Biofortified crop cultivars released from 2004 to 2020 (source: www.harvestplus.org).

The next breeding steps are the intermediate and final stage product development, which take place at both CGIAR centers and National Agricultural Research Systems (NARS), where breeding materials with significantly improved nutrient concentration coupled with equal or high agronomic performance over local control variety, as well as consumer-preferred quality, are developed. When promising high-yielding, high-nutrient lines are available, these are tested in multiple target environments along with commercial checks. Participatory variety selection (PVS) involving farmers and/or consumers is also practiced in few crops (for example in wheat; Velu et al., 2015). The best-performing lines are identified over multi-site testing for genotype by the assessment of environment (GxE) interactions and then submission to respective national varietal release committees for their release (orange boxes). The breeding process takes 6 to 10 years to complete depending on the crop, screening capacity, testing networks, and program turnaround. As a result of TB, HarvestPlus has supported programs and, together with its CIP partner, has released 393 biofortified varieties of 12 crops in 49 countries (Figure 2; Supplementary Table 1).

### TB: Progress and Variety Releases

The biofortified germplasm, varietal development, and release progress until 2016 was reviewed by Andersson et al. (2017). The following section briefly discusses the progress made in crop development and variety releases until 2020 for primary staple crops.

#### Zinc Wheat

Global wheat production was at a record high at 773 million metric tons in 2020–2021 (http://www.fao.org/faostat/en/#data/QCL; https://www.statista.com/statistics/267268/production-of-wheat-worldwide-since-1990/). Wheat provides ≥20% of daily dietary energy globally and is a good source of iron and zinc (Velu et al., 2017). A breeding target of an additional 12 ppm was set [(CAST (Council for Agricultural Science and Technology), 2020)]. Several studies have indicated that ample genetic variation in wheat exists for the success of a biofortified wheat breeding program. Hence, to start with, a breeding program was initiated in the International Maize and Wheat Improvement Center (CIMMYT). In particular, two key nurseries, namely, HarvestPlus Advanced (HPAN) and HarvestPlus Yield Trial (HPYT), bred at CIMMYT are routinely shared with NARS in India and Pakistan for varietal development (Virk et al., 2021). These nurseries are field-evaluated at multiple locations to shortlist candidate varieties possessing >6 ppm additional Zn concentration. Elite lines are then submitted to the varietal release programs in respective countries. Recently, sharing of early-generation segregating materials (F4/F5) has also been started for the fast-track development of locally adapted varieties for high Zn. The new varieties have 100% target increment for grain Zn concentration (37 ppm) and are agronomically at par with or superior to the popular wheat cultivars of South Asia (e.g., Zincol-2016 and Akbar-2019), indicating that there is no yield trade-off (Velu et al., 2012, 2015, 2021; Virk et al., 2021). The incorporation of resistance to major diseases, such as yellow rust and stem rust (Ug99), was also built into zinc wheat. Twenty-one biofortified wheat varieties have been released in eight countries (Andersson et al., 2017; Supplementary Table 1). In 2020, the release of six wheat varieties (namely Zinc Gahun-1, Himganga, Khumal-Shakti, Zinc Gahun-2, Bheri-Ganga, and Borlaug) in one attempt was a tremendous achievement in Nepal (https://www.cimmyt.org/funder_partner/harvestplus/). Several promising candidate varieties are in the varietal development and evaluation pipeline.

#### Zinc Rice

Rice is one of the megastaples in the world, particularly in Asia where 90% of global rice is grown and consumed (Khush and Virk, 2000), and its consumption is steadily increasing in West Africa. Just like wheat, 12 ppm additional zinc was set as a breeding target. Initial screening by the International Rice Research Institute (IRRI) found a concentration of zinc of up to 58 ppm (reviewed by Andersson et al., 2017). HarvestPlus is focusing to release and deliver biofortified rice varieties in Bangladesh, Indonesia, and India. Breeding programs at IRRI, the Bangladesh Rice Research Institute (BRRI), Indonesian Center for Rice Research (ICRR), and the NARS in India have developed germplasm in early- to late-development stages and elite lines to final products. In addition, zinc rice breeding pipelines were established at the International Center for Tropical Agriculture (CIAT) targeted at Latin America. HarvestPlus is focusing on developing inbred varieties and not hybrids, because there is limited acceptability of hybrids outside China. In all, 14 rice varieties have been released in seven countries (Andersson et al., 2017; Supplementary Table 1). HarvestPlus has been approached by several partners in Africa to test zinc rice materials from CIAT and IRRI and NARS in Asia.

#### Iron Pearl Millet

Pearl millet is a staple cereal for around 90 million people in the arid and semi-arid regions of Sub-Saharan Africa (SSA) and South Asia (SA). The breeding program for iron pearl millet is based at ICRISAT. The initial screening of pearl millet germplasm reported an up to 76 ppm iron concentration, which was sufficient to undertake a breeding program to enhance iron concentration by an additional 30 ppm (Bouis and Saltzman, 2017).

The targeted breeding for iron pearl millet aimed to identify improved open-pollinated varieties (OPVs), advanced breeding lines, and hybrid parents with a moderate to higher level of iron/zinc to develop and release biofortified cultivars. Dhanashakti, an OPV variety, is an improved version of ICTP 8203 for Fe density (by 9%), grain yield (by 11%) with higher fodder yield (by 13%), and similar to ICTP 8203 for other agronomic and adaptation traits (Rai et al., 2014). Several hybrids with more than 95% Fe target increment, with higher grain yield, have been identified (Govindaraj et al., 2019). However, the grain yield of these hybrids is not comparable with the highest-yielding commercial hybrid 86M86. A competitive yield gain can be achieved through diversifying the parents pool (*iniadi* sources) used in the initial stages of TB (Govindaraj et al., 2019). Emphasis is given to Fe trait introgression from donor sources to advanced seed and restorer parents through pedigree breeding. By introducing new breeding lines having a higher level of micronutrients and diversity among parentage, several promising hybrids have been developed and evaluated in a series of trials for possible testing and release in India.

Outside India, the West and Central Africa (WCA) region had the largest area under pearl millet in Africa (~15 m ha), and major staple food in this region. The most promising iron pearl millet OPVs are currently being tested on-farm in WCA. One OPV variety, namely, Chakti, has been released in Niger and gaining popularity in adjoining countries as well, owing to its early maturity. Several high-Fe lines from India were included in the WCA breeding program for fast-tracking the trait development and demonstrating the hybrid technology in the coming years. To date, 11 high-iron pearl millet cultivars (2 OPVs and 9 single cross hybrids) have been released in India and Niger (Andersson et al., 2017; Supplementary Table 1).

#### Iron Beans

Common bean is the most widely consumed food legume in Latin America and Eastern and Southern Africa. Beans have two growth habits, namely, bush and climbing, which are cultivated in low to mid-altitude and mid-to high-altitude areas, respectively. The initial screening of about 1,000 bean germplasm found up to 110 ppm Fe concentration in cultivated and wild species (Beebe et al., 2000). Existing variation was, therefore, enough to breed for a target increment level of 44 ppm Fe [for details, see Andersson et al., 2017; CAST (Council for Agricultural Science and Technology), 2020].

The breeding of biofortified beans is led by CIAT, who shares germplasm and advanced lines with NARS in several East and Southern African and South and Central American countries. Additionally, regional breeding programs based in Rwanda (Rwanda Agriculture Board, RAB) and the DRC (L'Institut National pour l'Etude et la Recherche Agronomique, INERA) are involved in developing high-Fe beans. In Rwanda and DRC, 10 and 19 varieties, respectively, were released until 2020. In Latin America, 23 high-iron bean varieties (20 bush and 3 climber types) have been released in eight countries (Andersson et al., 2017; Supplementary Table 1). Although gradual incremental Fe levels were achieved in beans, the recent releases hold a 100% iron target level. Important to note is that all these released bean varieties are resistant to major pests and diseases, have competitive yield, possess a range of colors and sizes, and have an acceptable cooking quality. In all, 69 bean varieties (43 bush and 26 climber types) have been released (Andersson et al., 2017; Supplementary Table 1). Crop development activities, strategies, and the varietal dissemination of biofortified beans in Rwanda are discussed in detail by Mulambu et al. (2017). To increase the adaptation of iron beans, future biofortified bean varieties should possess an adequate level of tolerance to drought and heat. On the other hand, the Fe bioavailability in beans should be increased by exploring low Phytic Acid (*lpa*) mutation breeding to optimize the *lpa* in future varieties.

#### Provitamin a Orange Sweet Potato

Sweet potato is widely consumed in SSA. Traditionally bred orange sweet potatoes (OSPs) containing PVA carotenoids were the first biofortified crop developed and released by the International Potato Center (CIP), HarvestPlus, and its partners. Plant breeders have produced several OSP varieties with PVA concentration exceeding the target level of 32 ppm. The proportion of beta carotene is >80% of the total carotenoid concentration in OSP (Woolfe, 1992). It is also established that the darker the orange color, the more beta-carotene present. This made breeding for high beta-carotene in OSP easier, and phenotypic selection for deep orange color was successful to breed OSP varieties exceeding the target level. Hence, the development and deployment of molecular markers are not necessary. HarvestPlus coordinates with NaCRRI and CIP in Uganda to ensure a strong breeding pipeline. Crop development activities, strategies, and the dissemination of biofortified sweet potato in SSA are discussed in detail by Low et al. (2017). One hundred and forty-six varieties of OSP (112 in Africa, 19 in Asia, and 15 in LAC) have been released in 28 countries (Supplementary Table 1).

#### Provitamin a Yellow Cassava

Cassava is a staple crop in most of tropical Africa, and it grows very well in poor soils. The screening of cassava germplasm at the CIAT found up to 19 ppm of PVA, exceeding the breeding target of 15 ppm (Andersson et al., 2017). Breeding programs for PVA cassava is based at CIAT and the International Institute of Tropical Agriculture (IITA). CIAT generates high PVA sources through rapid cycling and provides *in vitro* clones and populations to IITA and NARS in Nigeria and the Democratic Republic of Congo (DRC), for further breeding. These national research programs are the Nigerian (NRCRI) and National Root Crops Research Institute and the Institut National pour l'Etude et la Recherche Agronomiques (INERA) in the DRC. Nineteen varieties of yellow cassava have been released in Africa, and another 3 in Brazil (Andersson et al., 2017; Supplementary Table 1).

#### Provitamin a Orange Maize

Maize is very important for food security in SSA and Latin America. The initial screening of more than 1,500 maize germplasm accessions recorded a genetic variation of up to 19 ppm PVA, exceeding the PVA target of 15 ppm (Ortiz-Monasterio et al., 2007; Harjes et al., 2008; Menkir et al., 2015). PVA maize breeding programs at CIMMYT concentrated for tropical and mid-latitude, while IITA took over breeding for tropical lowlands. The Zambia Agriculture Research Institute (ZARI) is a national partner for PVA breeding. Both hybrid and open-pollinated (synthetic) biofortified varieties were bred. To date, in Africa, more than 65 PVA maize cultivars (synthetic, single-cross hybrids, and three-way hybrids) have been released in Cameroon, the DRC, Ghana, Malawi, Mali, Nigeria, Rwanda, Tanzania, Zambia, and Zimbabwe. A detailed review of activities and experiences with PVA delivery in Zambia is presented by Simpungwe et al. (2017). Five three-way hybrids fully meeting the PVA target level have been released in Ghana, Malawi, Tanzania, Zambia, and Zimbabwe (Supplementary Table 1). All the biofortified varieties combine high yield and acceptable grain quality traits.

#### Zinc Maize

In addition to breeding for PVA, both CIMMYT and IITA are also breeding for white maize with a higher Zn concentration. Up to 96 ppm Zn in tropical maize germplasm was found (Queiroz et al., 2011; Hindu et al., 2018). The initial focus has been on Latin American countries; however, several African nations, where white maize consumption is high, have also been included (Colombia, El Salvador, Guatemala, Honduras, Mexico, and Nicaragua; Benin, Ethiopia, Ghana, and Nigeria in Africa). Both QPM and non-QPM germplasm are being used extensively in making bi-parental and backcrosses for generating breeding materials (Prasanna et al., 2020). Both synthetic and hybrid varieties approaching the target increment (+12 ppm additional zinc) have been released. Eleven zinc maize varieties (seven OPVs, three three-way hybrids, and one single cross hybrid) have been released in five Latin American countries (Supplementary Table 1).

## TB and Marker-Aided Selection for FE, ZN, and PVA

Quantitative trait loci for grain zinc concentration have been mapped in wheat (Hao et al., 2014; Velu et al., 2014; Velu and Singh, 2019), rice (Stangoulis et al., 2007; Banerjee et al., 2010; Anuradha et al., 2012; Neelamraju et al., 2012; Norton et al., 2014; Swamy et al., 2018; Descalsota-Empleo et al., 2019; Calayugan et al., 2020), and maize (Hindu et al., 2018; Prasanna et al., 2020). Major quantitative trait loci (QTLs) for both iron and zinc concentration have been mapped in pearl millet (Kumar et al., 2016, 2018; Anuradha et al., 2017) and beans (Izquirdo et al., 2018). Metal transporter gene families in rice (Palmgren et al., 2008; Stomph et al., 2009) and pearl millet (Mahendrakar et al., 2020), which play an important role in Fe and Zn homeostasis, are also being investigated to enhance Zn concentration. However, all these QTLs are being validated in diverse genetic backgrounds, and none of these are being used routinely in varietal development breeding pipelines to enhance Zn concentration.

In cassava, two single-nucleotide polymorphism (SNP) markers in the exon region of phytoene synthase 2 (*psy2*) have been associated with yellow color. These two mutations are responsible for the generation of a more active enzyme; hence, they enhance total carotenoid production (das Araújo et al., 2021). High throughput molecular assays for these SNPs, along with phenotypic selection, could be performed to enhance carotenoid concentration in the breeding programs (Welsch et al., 2010; Ferguson et al., 2011; Rabbi et al., 2014).

In maize, however, two different genes within the carotenoid biosynthetic pathway, namely, *beta-carotene hydroxylase 1* (*CrtRB1*), which catalyzes the hydroxylation of β-carotene (BC) to *beta-cryptoxanthin* (BCX), and *lycopene epsilon cyclase* (*LcyE*), which converts lycopene into ζ-carotene and ultimately α-carotene (Harjes et al., 2008; Yan et al., 2010), have been targeted to increase PVA. Molecular assays for three functional polymorphisms within these two genes have been shown to more than double the BC concentration and at the same time reduce by up to 30% the ratio of alpha to beta carotenoids (Babu et al., 2013; Zunjare et al., 2018). More recently, marker-aided selection for PVA and MSV1 alleles is being carried out in the breeding program at CIMMYT (Prasanna et al., 2020).

## Mainstreaming (MS)

Targeted breeding, a time-tested breeding strategy, at CGIAR centers and NARS was instrumental in developing competitive biofortified varieties and improving the concept of high yield, and other agronomic traits could be combined with mineral and vitamin density, as has been described in the earlier sections. Nevertheless, to fulfill the ambitious target of HarvestPlus, which is to scale out to reach more than a billion people by 2030, CGIAR center breeding programs are aligned to address key sustainable development goals (SDGs) in the coming years. Therefore, all the germplasm and breeding lines being distributed by CGIAR centers for a specific crop, and submitted by NARS to varietal release committees, should be biofortified, assuring minimum target nutrient levels (i.e., 50% of breeding target). Taking advantage of the best agronomic characteristics of varieties and hybrids from all stakeholders, eventually, all or most varieties and hybrids are expected to be bred as higher-yielding biofortified varieties. A short-hand term that has been coined to describe this strategy is “mainstreaming.” In other words, mainstreaming refers to incorporating micronutrient traits into all germplasm and breeding pipelines targeted for production zones for which it constitutes a value addition. By integrating higher levels of key nutrients in all breeding lines without any counterproductive agronomic performance will lead to the development of offspring and varieties from the CG centers, and NARS pipelines are expected to be biofortified. For biofortification traits, in particular, minerals that remain stable, only minimal “maintenance breeding” would be required once mainstreaming is accomplished.

### Transition From Targeted Breeding to Mainstreaming

The targeted breeding approach was capitalized on traditional breeding approaches and to a certain extent, the preliminary application of marker-aided selection. Modernizing breeding programs are driven by advances in technology including genomic selection coupled with speed breeding and are likely to enhance mainstreaming process. In addition, establishment of several synergistic collaborative projects and platforms commended breeders for a “big push” for mainstreaming nutrition through simultaneous selection for micronutrients and all core traits of interest.

#### Technological Advances

Multivariate genomic selection for several traits coupled with speed breeding has been demonstrated by Watson et al. (2019) to accelerate genetic gain.

#### Enabling Environment

Several collaborative projects and platforms, such as the Accelerating Genetic Gains in Maize and Wheat (AGG; https://www.cimmyt.org/projects/agg/); the High Throughput Genotyping (HTPG) Project (http://cegsb.icrisat.org/high-throughput-genotyping-project-htpg/); the Genomic Open Breeding Informatics Initiative (GOBii; http://gobiiproject.org/); the Integrated Breeding Platform (http://www.integratedbreeding.net), and the CGIAR Excellence in Breeding Platform (EiB; https://excellenceinbreeding.org/) are providing third party genotyping services, innovative decision-making tools for modernization of breeding programs to accelerate genetic gain.

#### Changing Funding Scenarios

**Figure 3:**
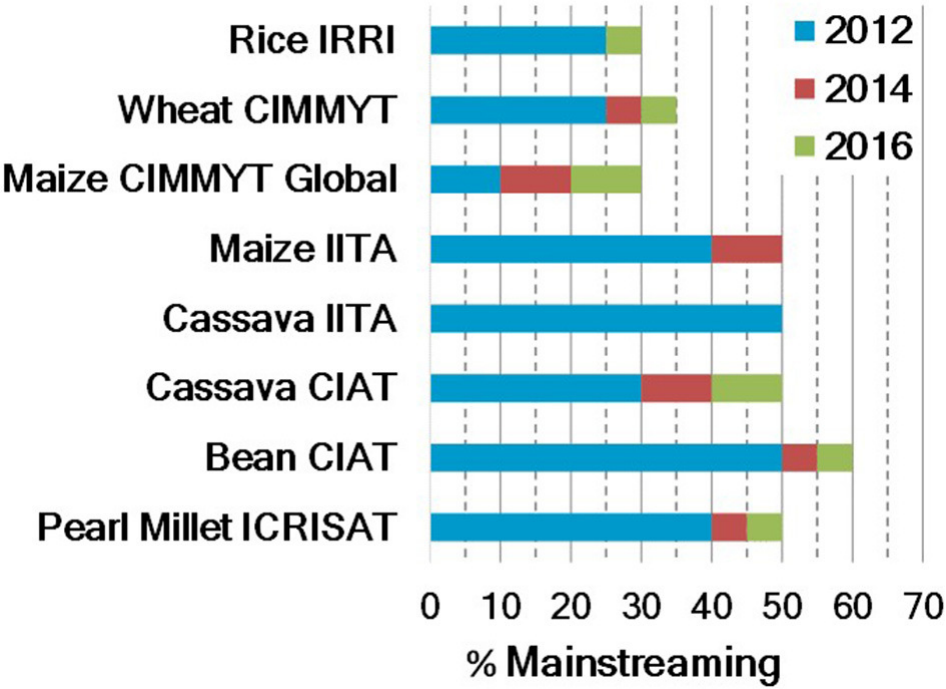
Percentage of mainstreaming effort at Consultative Group on International Agricultural Research (CGIAR) centers until 2016 [legend: blue (2012) indicates the cumulative % attained by the end of 2012; red and green, marginal additions by 2014 and 2016, respectively].

Donors initially funded TB and building on its success, they are starting to fund mainstreaming to expand the scope of biofortification. Mainstreaming is not a new concept. In the past, it was confined to TB; however, its meaning has expanded to the entire breeding pipeline because of mainstreaming efforts. While collaborators conducted TB, the crop development team of HarvestPlus working with crop leaders estimated the mainstreaming effort in the breeding programs of CG centers (Figure 3). Estimates were based on the proportion of the total program budget allocated to targeted biofortification breeding, the proportion of the number of crosses, and the proportion of biofortified lines in advanced yield trials or combinations. This model is giving more weightage to the investment to targeted biofortification breeding at each center. For instance, the proportion of the number of crosses and the proportion of biofortified lines in advanced yield trials or combinations that were delivered are proportional to the total investments to the given center. These proportions do not reflect certain parameters (e.g., competitiveness over commercial cultivars in that period), and certain target zones considered as irrelevant or breeding efforts for special projects may not have been included in the calculation of these figures. Overall, the amount of biofortified materials developed in each center is reflected, which will set a base to measure future mainstreaming efforts with the implementation of the newly proposed model (see next section).

In mainstreaming, incremental annual target nutrient increases for major crops vary between 2.5 and 3% per year. Much higher rates are necessary to achieve mainstreaming targets in a 10-year timeframe, before 2030 (Figure 3). Hence, additional crop development investments to accelerate this rate of genetic gains are crucial. We expect that it would take about 9–10 years until varieties from a mainstreaming “big push” with multivariate genomic selection for several quantitative traits of interest are released and adopted in target countries. Nevertheless, in this transition, TB is crucial to assure a constant flow of biofortified varieties until products from MS are available.

### Mainstreaming Capitalizes on Progress in Targeted Breeding

The experiences of the authors with TB have shown that to incorporate additional traits (e.g., Fe, Zn, and PVA) requires an additional effort and added human and financial resources, especially when the donor germplasm is from unadapted genetic backgrounds (landraces or wild species), and pre-breeding is often required. When the trait is transferred to established high-yielding germplasm and popular varieties *via* TB, their use as parents facilitates and accelerates MS as the next generation of micronutrient dense elite breeding lines and varieties used as parents are already in adapted genetic backgrounds. Hence, together with TB, mainstreaming is faster and efficient.

### Continuity of Targeted Breeding for Efficient Mainstreaming Progress

The achievements of HarvestPlus in reaching around 10 million farming households were driven by engaging in activities along the entire value chain, building critical partnerships on the supply and demand side in developing sustainable markets for seed and produce/products. Discontinuing TB may create genetic and breeding inefficiencies and eventually lead to dramatic consequences for the standards of the products and scaling of biofortification. TB is still not mature enough in some staples, and for target nutrients, the prospect for TB is equally high until mainstreaming demonstrates product delivery to meet nutritional standards and deliverables. For instance, stopping TB would disrupt activities in crop development and limit the availability of improved, competitive biofortified varieties. Hence, current versions of biofortified varieties would have to compete with newer non-biofortified varieties released by the CG centers, local NARS, and private seed companies until mainstreaming products are available. Furthermore, in the absence of biofortified varieties and their produce, several activities, such as advocacy, extension, continuous nutrition education, and marketing, would be reduced. Because of the limited short-term growth of biofortification, scaling would take much longer, for the mainstreaming products to reach farmer fields because of discontinuity. In the intervening years, there could be slower progress in reducing micronutrient deficiencies due to limited biofortification products, if any. For instance, minimum Fe and Zn standards were fixed in India's national pearl millet variety release policy in 2018 by the Indian Council of Agricultural Research (AICRP-PM, 2017). Such national policies are not in place in most staple crop variety guidelines. Most of the biotic stress tolerance and yield gains are being achieved by such a cultivar release policy at the regional level and globally. Having such a standard policy specific to crops may balance TB efforts and enhance mainstreaming efforts. To date, there is no report available on conserving biofortified germplasm, breeding lines, and hybrid parents (including the released biofortified varieties) in gene banks at CG centers.

**Figure 4:**
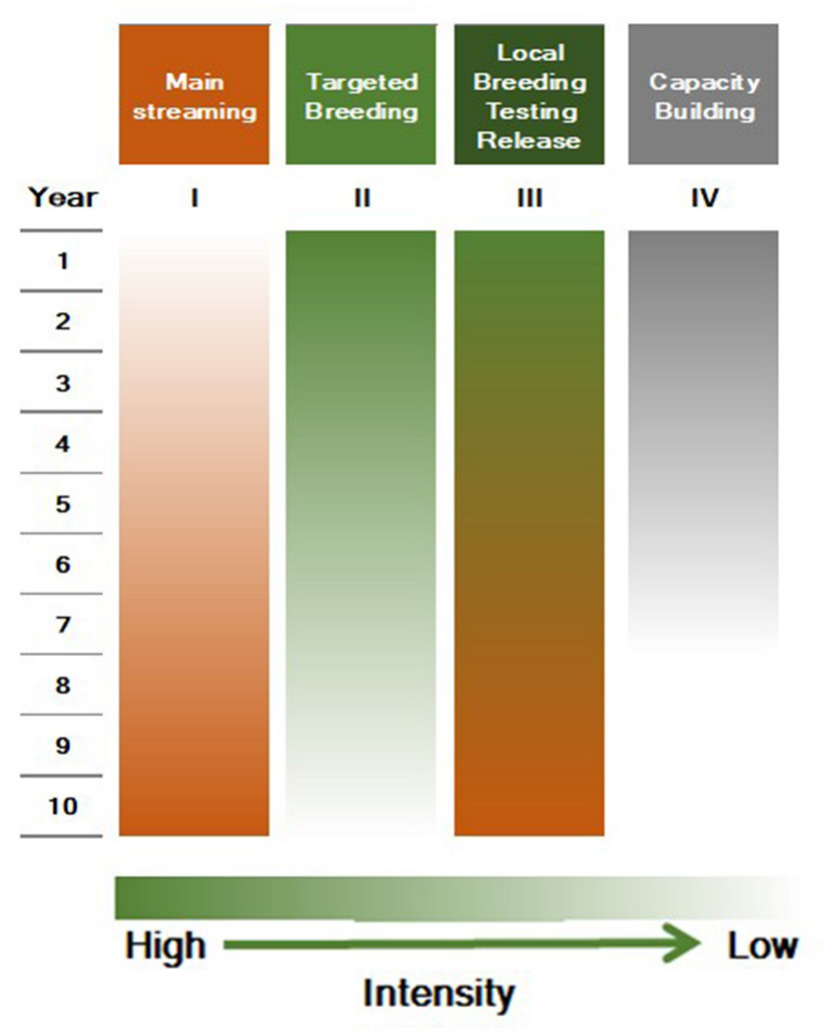
Proposed strategy and timeline for a gradual shift from targeted breeding to mainstreaming.

It is critical to invest in TB and mainstreaming is required until a sufficient number of biofortified parental lines are established in breeding centers and, more importantly, conserved in local and global gene banks (as global public goods). The proposed twin strategy along with timelines is outlined in Figure 4, and would enable the mainstreaming of biofortified products to address malnutrition significantly. In addition to the additional “big push” support for mainstreaming (I) to the CG Centers, HarvestPlus continues TB support to CG centers and NARS, and capacity building, for 5 years (II–IV) while monitoring the mainstreaming in CG centers. Given this scenario, HarvestPlus would gradually phase out funding crop development and thereafter may concentrate on an oversight and monitoring role. Collaborating CG centers and public and private sector NARS will absorb a decrease in TB funding after year 5. Mainstreaming is in its initial stages for wheat, rice, beans, cassava, and pearl millet at CIMMYT, IRRI, CIAT, and ICRISAT. Assured long-term funding is required for the sustainability of mainstreaming.

### New Role of HarvestPlus

HarvestPlus is continuously evolving its global and regional focus with a diverse working expert team. HarvestPlus, in consultation with various crop lead CG centers, has developed a set of mainstreaming indicators for wheat, rice, maize, beans, pearl millet, and cassava to monitor initial mainstreaming breeding progress. These first ever indicators (Tables 3, 4) are developed for self-pollinated and cross-pollinated crops. These primary indicators will continue to evolve and be customized for contributing to crop product profiles and varietal replacement. Therefore, HarvestPlus and EiB are driving the higher yields as well as higher nutrition staple food crops as part of the targeted genetic gains in the near future.

**Table 3:** Indicators for assessing and monitoring the transition to mainstreaming of biofortification in self-pollinating crops.

a) ***Percentage of crosses*** with biofortified germplasm vs. total number of crosses for each product profile
b.1) ***Percentage*** of biofortified lines vs. total number of breeding lines in testing at different evaluation stages for each product profile^*^: Stage 1 Breeding center Stage 2 Multilocation/target sites Stage 3 National trials
b.2) ***Performance*** of biofortified lines vis-à-vis non-biofortified lines (commercial checks) in testing ***at different evaluation stages*** for each product profile: Stage 1 Multilocation trials Stage 2 Advanced trials Stage 3 National variety trials
c.1) ***Percentage of yield trials*** established with biofortified germplasm vs. total yield trials (under same conditions of testing) for each product profile
c.2) ***Agronomic*****comp*****etitiveness*** of biofortified germplasm which constitutes a value proposition to farmers for each product profile e.g. yield advantage over commercial checks, resistance to diseases (rust and foliar diseases), stress tolerance etc.
c.3) ***End-use qualitycompetitiveness*** of biofortified germplasm which constitutes a value proposition to farmers/processors/traders for each product profile (advantage over popular varieties) e.g., milling quality, cooking quality (flat bread/steamed bread) and organoleptic properties
c.4) Rate of expected or realized ***genetic gain*** for micronutrient (MN) trait, yield and key essential traits for each product profile
d) Percentage of ***budget allocation*** specific to biofortification breeding vs. total breeding budget
e) Frequency or % of ***favorable markers/alleles used to predict the performance of MN trait*** in breeding populations for each product profile

* *Product profiles are established in each CGIAR breeding center (for elite germplasm/parents), and similar profiles are being developed in National Agricultural Research System (NARS) centers (for variety replacement)*.

**Table 4:** Indicators for assessing and monitoring the transition to mainstreaming of biofortification in cross-pollinating crops.

a) ***Percentage of crosses/test crosses*** with biofortified germplasm vs. total number of crosses/test crosses for each product profile
b.1) ***Percentage*** of biofortified OPVs/Synthetics/hybrids vs. total number of OPVs/Synthetics/hybrids in testing at different evaluation stages for each product profile^*^: Stage 1 Breeding center Stage 2 Multilocation/target sites Stage 3 National trials
b.2) ***Performance*** of biofortified OPVs/Synthetics/hybrids vis-à-vis non-biofortified OPVs/Synthetics/hybrids (commercial checks) in testing ***at different evaluation stages*** for each product profile: Stage 1 Multilocation trials Stage 2 Advanced trials Stage 3 National hybrid/variety trials
c.1) ***Percentage of yield trials*** established with biofortified germplasm vs. total yield trials (under same conditions of testing) for each product profile
c.2) ***Agronomic*****comp*****etitiveness*** of biofortified germplasm which constitutes a value proposition to farmers for each product profile (advantage over commercial checks) e.g., yield advantage over commercial checks, resistance to diseases (Striga, MLN), stress tolerance etc.
c.3) ***End-use qualitycompetitiveness*** of biofortified germplasm which constitutes a value proposition to farmers/processors/traders for each product profile (advantage over popular varieties) e.g., milling performance, processing quality, sensory acceptance of the target populations
c.4) Rate of expected or realized ***genetic gain*** for micronutrient (MN) trait, yield and key essential traits for each product profile
d) Percentage of ***budget allocation*** specific to biofortification breeding vs. total breeding budget
e) Frequency or % of ***favorable markers/alleles used to predict the performance of MN trait*** in breeding populations for each product profile

* *Product profiles are established in each CGIAR breeding center (for elite germplasm/parents), and similar profiles are being developed in NARS centers (for hybrid/variety replacement)*.

#### Mainstreaming and Product Profiles

The excellence in breeding (EiB) program of the CGIAR signified product profiles to focus on the breeding of crop varieties that would replace existing popular varieties on the market, considering market knowledge from millers, traders and consumers, and other considerations such as gender. Product profile describes a variety with the essential characteristics to replace as well as correct any defects (e.g., biotic stress tolerance and nutrition) of largely cultivated varieties. It also serves as a commitment by breeders to stakeholders. HarvestPlus is working very closely with the EiB module lead and crop lead centers to ensure that essential vitamins and micronutrient traits are embedded as a core trait in CGIAR and NARS crop product profiles. HarvestPlus is also working on similar lines with crop breeders in CG and NARS partner centers to include nutritional traits under “must-have traits” and “nice to have traits,” depending on the stage of trait deployment in the respective breeding program. Crops such as wheat, rice, pearl millet, beans, and cassava should make it as mandatory traits, since nutrient standards and desired levels are achieved in a large set of parental pools and pipelines developed in CGIAR and NARS breeding centers; whereas other crops may be classified as value-added or market traits, which are required to be incorporated in the next 5–6 years and then upgraded to must-have trait provided critically reviewed and endorsed by the product designing team. Therefore, stringent selection for Fe, Zn, PVA should be exercised in addition to agronomic traits in the mainstream breeding. Including these nutrients traits in product profiles would ensure genetic gains for these traits along with competitive yields in the future (https://repo.mel.cgiar.org/handle/20.500.11766/10236).

### The Key Role of NARS in Mainstreaming

Any discussion *vis-à-vis* TB and mainstreaming must consider the key role of NARS in the biofortified variety testing and release process. NARS and private seed companies regularly introduce germplasm from CGIAR centers for adaptive testing and subject it to additional breeding. Once adaptive promising germplasm is identified among introduced and improved elite lines, NARS or private seed companies will submit candidates to national variety release committees for official testing and the formal release and notification of varieties. CGIAR centers submit promising germplasm through NARS partners. NARS breeding programs and local seed companies are also responsible for the production and marketing of various classes of seed, and a large quantity of certified seed is required to fulfill demand. In some NARS where well R and D facilities and established seed systems do not exist, HarvestPlus needs to continue to support adaptive multi-location trials, breeding, and release of biofortified varieties. While MS continues at the CGIAR centers, adaptive GxE trials and the release of new biofortified varieties must continue to be undertaken by NARS.

### Capacity Building at CG Centers and NARS

The established TB improved biofortification breeding strategies, sampling methods, and phenotyping tools. Capitalizing on established novel techniques during the inception of biofortification mainstreaming is essential. Training and capacity building in high-throughput micronutrient screening by XRF and NIRS and with biofortification breeding methods at CGIAR and NARS have been essential for the success of mainstreaming. Breeding programs, as well as national variety release systems, must be able to quantify micronutrients to include Fe, Zn, or PVA as a nutritional trait in variety development and the release process. Since its inception, HarvestPlus, in partnership with Flinders University and other centers of excellence, has continued to lead an ongoing effort in micronutrient phenotyping capacity building and technical support with standardization, proficiency testing, equipment upgrades, training, and quality assurance. HarvestPlus has established several XRF labs across the world (https://www.harvestplus.org/knowledge-market/in-the-news/xrf-machines-innovative-technology-help-breed-nutritious-crops). These are key accelerators for successful biofortified verities as part of TB. Lab services are being extended for need-based analytical support to various stakeholders. Continuing future support for these activities by HarvestPlus and its partners is essential for the success of biofortification mainstreaming at CGIAR centers, NARS, and private sector breeding centers.

## Critical Elements for Successful Mainstreaming

Biofortification breeding addressed three important questions to scale up; (i) Is breeding for high nutrient content scientifically feasible? (ii) Will farmers adopt biofortified varieties? (iii) Will the varieties meet consumer acceptability to adopt and consume regularly? All the feasibilities were demonstrated by a large number of variety releases by TB and its household consumption in target countries. Stepping to mainstreaming requires the following three critical elements for the success of mainstreaming efforts (Bouis and Saltzman, 2017). This applies equally to the products developed from TB and mainstreaming. First, to increase the supply, require agricultural research institutions commitment to include Fe, Zn, and PVA traits along with core breeding traits; national or regional varietal release committees recommend minimum levels of Fe, Zn, and PVA as mandatory for varietal testing and release criteria. It is noteworthy that few governments such as India, China, and Brazil have allocated their resources to include biofortification in their R4D portfolio. In 2018, the All India Coordinated Pearl Millet Improvement Project (AICRP-PM) of the Indian Council of Agricultural Research (ICAR) made minimum standards for Fe (42 ppm) and Zn (32 ppm) requirements for national testing and approval of cultivar release in India. Second, policymakers must recognize the significant impact of biofortification to address nutritional insecurity. Substantial progress has already been made in integrating biofortification into regional and national policies. Several governments, such as India, China, Brazil, Indonesia, Bangladesh, Malawi, Nigeria, Pakistan, Colombia, Panama, Rwanda, Zambia, and Uganda, have endorsed biofortification. Scaling Up Nutrition (SUN), Global Alliance for Improved Nutrition (GAIN), the Comprehensive Africa Agriculture Development Program (CAADP) movements of the African Union are building an enabling environment for biofortification**.** Third, demand creation is essential through various nutritional education/awareness campaigns, for high mineral and vitamin contents in their staple food and food products.

## Current Status and Prospects of Mainstreaming

A gradual transition and coexistence of both strategies shall continue until a sufficient number of biofortified parental lines are established and varieties are released through mainstreaming. In many countries, both public and private sector seed companies rely on CGIAR-developed breeding materials, so mainstreaming will ensure the integration of biofortified traits into competitive varieties and hybrids developed by private companies and the public sector. The mainstreaming strategy has just been initiated in select CGIAR centers namely, CIMMYT, IRRI, ICRISAT, IITA, and CIAT. New CGIAR breeding strategies are likely to increase applications of various novel genomic tools and techniques, scaling biofortification from targeted to mainstreaming. HarvestPlus is working closely with strategic partners to develop/monitor crop strategies, and monitoring standards for CGIAR and NARS centers. For instance, combining genomic selection and speed breeding in wheat is the most advanced mainstreaming program. Several academic studies have advocated genomic selection to accelerate genetic gain for the improvement of zinc content (Zhang et al., 2015; Cao et al., 2017; Yuan et al., 2019, Jighly et al., 2019). Moderate to high genomic prediction accuracies have been reported in maize (.35 to.65; Prasanna et al., 2020) and wheat (0.33 to 0.69; Velu et al., 2016) for zinc content across different types of populations and genotyping platforms. The grain Zn content along with other core traits in maize, wheat, and other crops could be improved by employing marker-aided selection for validated haplotypes to enrich segregants with favorable alleles in the earlier generations followed by multivariate genomic selection at either earlier or later generations along with speed breeding.

However, research is still ongoing on best ways to use genomic selection that result in most accurate predictions and ultimately reduce selection cycle time. At the same time, CIMMYT has started putting together an optimizing genomic selection mainstreaming strategy incorporating limited speed breeding in wheat, while ICRISAT has just established a speed breeding platform to accelerate mainstreaming for pearl millet. Joining forces from different projects such as AGG and EiB breeding scheme optimization is ongoing at CIMMYT [e.g., Rapid Bulk Generation Advancement' (RBGA); Rapid Cycling Recurrent Selection (RCRS)]. Double haploidy, instead of speed breeding, for line advancement is a method of choice in maize. The integration of multivariate GS and speed breeding is essential to accelerate genetic gains in mainstreaming; however, it is too early to conclude its success in developing and delivering successful biofortified varieties. In the future, HarvestPlus and EiB can work closely to catalyze the mainstreaming of nutrition and networking services to CGIAR and NARS centers besides modernizing the breeding programs. Therefore, TB and mainstreaming would continue until a sufficient number of biofortified parental lines are established and competitive biofortified varieties developed through mainstreaming pipelines are released and adopted. Alternatively, the active involvement and commitments from private sector breeding organizations for biofortified products and seed marketing may reduce the overall time frame of mainstreaming.

## Author Contributions

PV and WP designed the review. PV, MA, JA, MG, and WP wrote the manuscript. PV and MG revised the manuscript. All the authors read and approved the final manuscript.

## Funding

This work was supported by BMGF HarvestPlus 3: Grant number OPP1019962.

## Conflict of Interest

The authors declare that the research was conducted in the absence of any commercial or financial relationships that could be construed as a potential conflict of interest.

## Publisher's Note

All claims expressed in this article are solely those of the authors and do not necessarily represent those of their affiliated organizations, or those of the publisher, the editors and the reviewers. Any product that may be evaluated in this article, or claim that may be made by its manufacturer, is not guaranteed or endorsed by the publisher.
