# Supplementary material for: Transition From Targeted Breeding to Mainstreaming of Biofortification Traits in Crop Improvement Programs
Source: Front Plant Sci. 2021 Sep 14;12:703990. doi: 10.3389/fpls.2021.703990 (PMC8477801; doi:10.3389/fpls.2021.703990)
Supplement: Supplementary file 1 [file Table_1.pdf]

Supplementary table 1. Biofortified crops and varieties released in various countries.

S.No.	Crop	Country	Biofortified Crop Name	Variety Name	Year Release	Primary Target MN	Primary MN Concentration (ppm)	Remarks
1	Banana/Plantain	Burundi	Vitamin A Banana/Plantain	Muracho	2021	Vitamin A	154.6 (FW, Stage 5)	fast-track
2	Banana/Plantain	Burundi	Vitamin A Banana/Plantain	Apantu	2014	Vitamin A	100.6 (FW, Stage 5)	fast-track
3	Banana/Plantain	Burundi	Vitamin A Banana/Plantain	Bira	2021	Vitamin A	106.3 (FW, Stage 5)	fast-track
4	Banana/Plantain	Burundi	Vitamin A Banana/Plantain	Pelipita	2021	Vitamin A	17.4 (FW, Stage 5)	fast-track
5	Banana/Plantain	Burundi	Vitamin A Banana/Plantain	To'o	2014	Vitamin A	77.6 (FW, Stage 5)	fast-track
6	Banana/Plantain	Burundi	Vitamin A Banana/Plantain	Lai	2021	Vitamin A	105.1 (FW, Stage 5)	fast-track
7	Banana/Plantain	DR Congo	Vitamin A Banana/Plantain	Muracho	2019	Vitamin A	154.6 (FW, Stage 5)	fast-track
8	Banana/Plantain	DR Congo	Vitamin A Banana/Plantain	Pisang Papan	2019	Vitamin A	108.1 (FW, Stage 5)	fast-track
9	Banana/Plantain	DR Congo	Vitamin A Banana/Plantain	Apantu	2014	Vitamin A	100.6 (FW, Stage 5)	fast-track
10	Banana/Plantain	DR Congo	Vitamin A Banana/Plantain	Bira	2014	Vitamin A	106.3 (FW, Stage 5)	fast-track
11	Banana/Plantain	DR Congo	Vitamin A Banana/Plantain	Pelipita	2014	Vitamin A	17.4 (FW, Stage 5)	fast-track
12	Banana/Plantain	DR Congo	Vitamin A Banana/Plantain	To'o	2014	Vitamin A	77.6 (FW, Stage 5)	fast-track
13	Banana/Plantain	DR Congo	Vitamin A Banana/Plantain	Lahi	2014	Vitamin A	105.1 (FW, Stage 5)	fast-track
14	Beans	Bolivia	Iron Beans	Fortaleza	2009	Iron	87	
15	Beans	Brazil	Iron Beans	BRS Agreste	2009	Iron	78.8	
16	Beans	Brazil	Iron Beans	BRS 9435 Cometa	2007	Iron	80	
17	Beans	Brazil	Iron Beans	BRS Pontal	2004	Iron	77	
18	Beans	Burundi	Iron Beans	MAC 44	2015	Iron	TBD	
19	Beans	Burundi	Iron Beans	RWV 1129	2015	Iron	TBD	
20	Beans	Burundi	Iron Beans	RWR 2245	2015	Iron	76	
21	Beans	Burundi	Iron Beans	RWR 2154	2015	Iron	71	
22	Beans	Colombia	Iron Beans	Corpoica Rojo 39	2015	Iron	86	
23	Beans	Colombia	Iron Beans	Corpoica Rojo 43	2015	Iron	95	
24	Beans	Colombia	Iron Beans	BIO-101	2016	Iron	83	
25	Beans	Colombia	Iron Beans	BIO-107	2016	Iron	82	
26	Beans	Colombia	Iron Beans	BIO-102	2019	Iron	86	
27	Beans	DR Congo	Iron Beans	COD MLB 001	2012	Iron	66	fast-track
28	Beans	DR Congo	Iron Beans	HM 21-7	2012	Iron	62	fast-track
29	Beans	DR Congo	Iron Beans	RWR 2245	2011	Iron	66	
30	Beans	DR Congo	Iron Beans	COD MLV 059	2012	Iron	84	
31	Beans	DR Congo	Iron Beans	VCB 81013	2012	Iron	69	fast-track
32	Beans	DR Congo	Iron Beans	Pigeon Vert	2012	Iron	80	fast-track
33	Beans	DR Congo	Iron Beans	PVA 1438	2013	Iron	79	
34	Beans	DR Congo	Iron Beans	Nain de Kyondo	2013	Iron	76	
35	Beans	DR Congo	Iron Beans	COD MLB 032	2013	Iron	76	
36	Beans	DR Congo	Iron Beans	Cuarentino 0817	2013	Iron	100	
37	Beans	DR Congo	Iron Beans	NUA 100	2016	Iron	78	
38	Beans	DR Congo	Iron Beans	MNC 488-2	2016	Iron	79	
39	Beans	DR Congo	Iron Beans	NUV 119-4	2016	Iron	72	
40	Beans	DR Congo	Iron Beans	RWR 2154	2011	Iron	71	
41	Beans	DR Congo	Iron Beans	M 211	2012	Iron	77	
42	Beans	DR Congo	Iron Beans	Namulenga	2013	Iron	76	
43	Beans	DR Congo	Iron Beans	Ecapan 021	2013	Iron	72	
44	Beans	DR Congo	Iron Beans	Nyiramuhondo	2016	Iron	TBD	
45	Beans	DR Congo	Iron Beans	NUA 99	2018	Iron	TBD	
46	Beans	El Salvador	Iron Beans	CENTA FerromÃ¡s	2011	Iron	77	
47	Beans	Guatemala	Iron Beans	ICTA Superchiva-ACM	2011	Iron	TBD	
48	Beans	Guatemala	Iron Beans	ICTA Peten-ACM	2010	Iron	TBD	
49	Beans	Guatemala	Iron Beans	ICTA Chorti-ACM	2017	Iron	95	
50	Beans	Honduras	Iron Beans	Honduras Nutritivo	2016	Iron	71	
51	Beans	Nicaragua	Iron Beans	INTA Ferroso	2014	Iron	79	
52	Beans	Nicaragua	Iron Beans	INTA Nutritivo	2012	Iron	70	
53	Beans	Nicaragua	Iron Beans	INTA BIOF100	2017	Iron	80	
54	Beans	Nicaragua	Iron Beans	INTA BIODOR	2017	Iron	76	
55	Beans	Nicaragua	Iron Beans	INTA Rojo Bio-Apante	2019	Iron	91	
56	Beans	Panama	Iron Beans	IDIAP NUA 24	2012	Iron	78	
57	Beans	Panama	Iron Beans	IDIAP NUA 27	2012	Iron	TBD	
58	Beans	Panama	Iron Beans	IDIAP P-09-11	2018	Iron	87	
59	Beans	Panama	Iron Beans	IDIAP P-13-38	2018	Iron	78	
60	Beans	Rwanda	Iron Beans	MAC 44	2010	Iron	78	
61	Beans	Rwanda	Iron Beans	RWV 1129	2010	Iron	77	
62	Beans	Rwanda	Iron Beans	CAB 2	2010	Iron	81	
63	Beans	Rwanda	Iron Beans	RWR 2245	2010	Iron	76	
64	Beans	Rwanda	Iron Beans	RWR 2154	2010	Iron	71	
65	Beans	Rwanda	Iron Beans	RWV 3316	2012	Iron	87	
66	Beans	Rwanda	Iron Beans	RWV 3006	2012	Iron	78	
67	Beans	Rwanda	Iron Beans	RWV 3317	2012	Iron	74	
68	Beans	Rwanda	Iron Beans	MAC 42	2012	Iron	91	
69	Beans	Rwanda	Iron Beans	RWV 2887	2012	Iron	85	
70	Beans	Tanzania	Iron Beans	Selian 14	2018	Iron	85	
71	Beans	Tanzania	Iron Beans	Selian 15	2018	Iron	81	
72	Beans	Tanzania	Iron Beans	TARIBEAN 2	2020	Iron	87.2	
73	Beans	Tanzania	Iron Beans	TARIBEAN 4	2020	Iron	78.3	
74	Beans	Tanzania	Iron Beans	TARIBEAN 5	2020	Iron	90.5	
75	Beans	Tanzania	Iron Beans	TARI 19	2020	Iron	71	
76	Beans	Uganda	Iron Beans	Roba 1	2013	Iron	64	
77	Beans	Uganda	Iron Beans	NARO Bean 4C	2016	Iron	78	
78	Beans	Uganda	Iron Beans	NARO Bean 2	2016	Iron	76	
79	Beans	Uganda	Iron Beans	NARO Bean 1	2016	Iron	71	
80	Beans	Uganda	Iron Beans	NARO Bean 3	2016	Iron	70	
81	Beans	Uganda	Iron Beans	NARO Bean 5C	2016	Iron	67	
82	Beans	Zimbabwe	Iron Beans	Jasmine	2019	Iron	86	
83	Cassava	Brazil	Vitamin A Cassava	BRS Jari	2009	Vitamin A	7.7	
84	Cassava	Brazil	Vitamin A Cassava	BRS Gema de Ovo	2005	Vitamin A	3.4	
85	Cassava	Brazil	Vitamin A Cassava	BRS Dourada	2005	Vitamin A	4.5	
86	Cassava	Cameroon	Vitamin A Cassava	I070593	2019	Vitamin A	11.4	
87	Cassava	DR Congo	Vitamin A Cassava	Kindisa	2008	Vitamin A	7.6	
88	Cassava	DR Congo	Vitamin A Cassava	Vimpi	2017	Vitamin A	10.4	
89	Cassava	DR Congo	Vitamin A Cassava	Lumonu	2017	Vitamin A	9.5	
90	Cassava	DR Congo	Vitamin A Cassava	Mukoleshi	2017	Vitamin A	7.9	
91	Cassava	Ghana	Vitamin A Cassava	Nyonku agbeli	2019	Vitamin A	9.6	
92	Cassava	Ghana	Vitamin A Cassava	Tetteh bankye	2019	Vitamin A	6.9	
93	Cassava	Ghana	Vitamin A Cassava	Kpornu agbeli	2019	Vitamin A	7.9	
94	Cassava	Ghana	Vitamin A Cassava	CRI-Manu	2020	Vitamin A	10.4	
95	Cassava	Ghana	Vitamin A Cassava	â€‹CRI-Opemfo Bankye	2020	Vitamin A	7.8	
96	Cassava	Ghana	Vitamin A Cassava	CRI-Lizzy	2020	Vitamin A	TBD	

**97**	Cassava	Ghana	Vitamin A Cassava	CRI-Mmofra Bankye	2020	Vitamin A	12.7	
**98**	Cassava	Nigeria	Vitamin A Cassava	UMUCASS 44	2014	Vitamin A	10.8	
**99**	Cassava	Nigeria	Vitamin A Cassava	UMUCASS 37	2011	Vitamin A	7.4	
**100**	Cassava	Nigeria	Vitamin A Cassava	UMUCASS 36	2011	Vitamin A	6.9	
**101**	Cassava	Nigeria	Vitamin A Cassava	UMUCASS 45	2014	Vitamin A	11.4	
**102**	Cassava	Nigeria	Vitamin A Cassava	UMUCASS 46	2014	Vitamin A	10.9	
**103**	Cassava	Nigeria	Vitamin A Cassava	UMUCASS 38	2011	Vitamin A	7.8	
**104**	Cassava	Sierra Leone	Vitamin A Cassava	Slicass 12	2014	Vitamin A	8.8	
**105**	Cowpea	Brazil	Iron Cowpea	BRS AracÃª	2009	Iron	61.7	
**106**	Cowpea	Brazil	Iron Cowpea	BRS Tumucumaque	2009	Iron	60.6	
**107**	Cowpea	Brazil	Iron Cowpea	BRS Xiquexique	2008	Iron	77.4	
**108**	Cowpea	India	Iron Cowpea	Pant Lobia-2	2010	Iron	100	
**109**	Cowpea	India	Iron Cowpea	Pant Lobia-1	2008	Iron	82	
**110**	Cowpea	India	Iron Cowpea	Pant Lobia-4	2014	Iron	52	
**111**	Cowpea	India	Iron Cowpea	Pant Lobia-3	2013	Iron	97	
**112**	Cowpea	India	Iron Cowpea	Pant Lobia-5	2015	Iron	66	
**113**	Cowpea	India	Iron Cowpea	Pant Lobia-7	2019	Iron	TBD	
**114**	Lentils	Bangladesh	Iron Lentil	Barimasur-7	2012	Iron	61	
**115**	Lentils	Bangladesh	Iron Lentil	Barimasur-8	2015	Iron	60	
**116**	Lentils	Bangladesh	Iron Lentil	Barimasur-4	2010	Iron	51	fast-track
**117**	Lentils	Bangladesh	Iron Lentil	Barimasur-5	2010	Iron	59	fast-track
**118**	Lentils	Bangladesh	Iron Lentil	Barimasur-6	2010	Iron	63	fast-track
**119**	Lentils	India	Iron Lentil	L 4704	2013	Iron	74	
**120**	Lentils	Nepal	Iron Lentil	ILL 7723	2013	Iron	62	
**121**	Lentils	Nepal	Iron Lentil	Rasuwa black	2020	Iron	TBD	
**122**	Lentils	Syria	Iron Lentil	Idlib-2	2010	Iron	TBD	fast-track
**123**	Lentils	Syria	Iron Lentil	Idlib-3	2010	Iron	TBD	fast-track
**124**	Maize (VitA)	Brazil	Vitamin A Maize	BRS 4104	2015	Vitamin A	8	
**125**	Maize (VitA)	Cameroon	Vitamin A Maize	PVA SYN 6 F2	2018	Vitamin A	7	
**126**	Maize (VitA)	Cameroon	Vitamin A Maize	PVA SYN 13 F2	2018	Vitamin A	9	
**127**	Maize (VitA)	DR Congo	Vitamin A Maize	Sam 4 Vita	2014	Vitamin A	8.8	
**128**	Maize (VitA)	DR Congo	Vitamin A Maize	Muibaki 3	2016	Vitamin A	10	
**129**	Maize (VitA)	DR Congo	Vitamin A Maize	Muibaki 2	2016	Vitamin A	7	
**130**	Maize (VitA)	DR Congo	Vitamin A Maize	Muibaki 1	2016	Vitamin A	6	
**131**	Maize (VitA)	DR Congo	Vitamin A Maize	GV664A	2014	Vitamin A	7.2	
**132**	Maize (VitA)	DR Congo	Vitamin A Maize	GV665A	2014	Vitamin A	7.8	
**133**	Maize (VitA)	DR Congo	Vitamin A Maize	LY1001-14	2017	Vitamin A	9.2	
**134**	Maize (VitA)	DR Congo	Vitamin A Maize	PVA SYN 10-F2	2017	Vitamin A	TBD	
**135**	Maize (VitA)	DR Congo	Vitamin A Maize	PVA SYN 18-F2	2017	Vitamin A	TBD	
**136**	Maize (VitA)	Ghana	Vitamin A Maize	CSIR-CRI Honampa	2012	Vitamin A	7	
**137**	Maize (VitA)	Ghana	Vitamin A Maize	CSIR-CRI Odomfo	2012	Vitamin A	6	
**138**	Maize (VitA)	Ghana	Vitamin A Maize	CSIR-CRI Owanwa	2012	Vitamin A	6	
**139**	Maize (VitA)	Ghana	Vitamin A Maize	AhoÉ”dzin	2015	Vitamin A	9.3	
**140**	Maize (VitA)	Ghana	Vitamin A Maize	Dzifoo	2015	Vitamin A	11.3	
**141**	Maize (VitA)	Ghana	Vitamin A Maize	AhoÉ”fÉ›	2015	Vitamin A	9.2	
**142**	Maize (VitA)	Ghana	Vitamin A Maize	CRI-Nkwagye	2019	Vitamin A	14.8	
**143**	Maize (VitA)	Ghana	Vitamin A Maize	CRI-Abebe	2019	Vitamin A	14	
**144**	Maize (VitA)	Malawi	Vitamin A Maize	MH39A	2016	Vitamin A	12	
**145**	Maize (VitA)	Malawi	Vitamin A Maize	MH40A	2016	Vitamin A	10	
**146**	Maize (VitA)	Malawi	Vitamin A Maize	MH42A	2016	Vitamin A	14.9	
**147**	Maize (VitA)	Malawi	Vitamin A Maize	MH43A	2016	Vitamin A	9.3	
**148**	Maize (VitA)	Malawi	Vitamin A Maize	MH44A	2017	Vitamin A	11	
**149**	Maize (VitA)	Malawi	Vitamin A Maize	MH46A	2018	Vitamin A	TBD	
**150**	Maize (VitA)	Malawi	Vitamin A Maize	MH45A	2018	Vitamin A	TBD	
**151**	Maize (VitA)	Malawi	Vitamin A Maize	MH47A	2018	Vitamin A	TBD	
**152**	Maize (VitA)	Malawi	Vitamin A Maize	MH48A	2018	Vitamin A	TBD	
**153**	Maize (VitA)	Malawi	Vitamin A Maize	MH49A	2018	Vitamin A	TBD	
**154**	Maize (VitA)	Mali	Vitamin A Maize	Nafama	2012	Vitamin A	7	
**155**	Maize (VitA)	Mali	Vitamin A Maize	Abebe	2016	Vitamin A	8	
**156**	Maize (VitA)	Mali	Vitamin A Maize	Duba	2016	Vitamin A	9	
**157**	Maize (VitA)	Mali	Vitamin A Maize	Kodialan	2016	Vitamin A	9	
**158**	Maize (VitA)	Mali	Vitamin A Maize	Dakan	2016	Vitamin A	7	
**159**	Maize (VitA)	Nigeria	Vitamin A Maize	Sammaz 38	2013	Vitamin A	5.9	
**160**	Maize (VitA)	Nigeria	Vitamin A Maize	Sammaz 39	2013	Vitamin A	6.7	
**161**	Maize (VitA)	Nigeria	Vitamin A Maize	Ife Hyb 3	2012	Vitamin A	8	
**162**	Maize (VitA)	Nigeria	Vitamin A Maize	Ife Hyb 4	2012	Vitamin A	7.8	
**163**	Maize (VitA)	Nigeria	Vitamin A Maize	Sammaz 43	2015	Vitamin A	7	
**164**	Maize (VitA)	Nigeria	Vitamin A Maize	Sammaz 44	2015	Vitamin A	7.8	
**165**	Maize (VitA)	Nigeria	Vitamin A Maize	Sammaz 49	2016	Vitamin A	8.2	
**166**	Maize (VitA)	Nigeria	Vitamin A Maize	Sammaz 52	2017	Vitamin A	8.6	
**167**	Maize (VitA)	Nigeria	Vitamin A Maize	SAMMAZ 59	2020	Vitamin A	16.3	
**168**	Maize (VitA)	Nigeria	Vitamin A Maize	SAMMAZ 60	2020	Vitamin A	15.53	
**169**	Maize (VitA)	Rwanda	Vitamin A Maize	RAHA03	2017	Vitamin A	TBD	
**170**	Maize (VitA)	Rwanda	Vitamin A Maize	RAHA04	2017	Vitamin A	TBD	
**171**	Maize (VitA)	Rwanda	Vitamin A Maize	RAHA02	2017	Vitamin A	TBD	
**172**	Maize (VitA)	Tanzania	Vitamin A Maize	Meru VAH 517	2016	Vitamin A	14.9	
**173**	Maize (VitA)	Tanzania	Vitamin A Maize	Meru VAH 519	2016	Vitamin A	7.8	
**174**	Maize (VitA)	Zambia	Vitamin A Maize	GV665A	2012	Vitamin A	7.8	
**175**	Maize (VitA)	Zambia	Vitamin A Maize	GV662A	2012	Vitamin A	4.7	
**176**	Maize (VitA)	Zambia	Vitamin A Maize	GV664A	2012	Vitamin A	7.2	
**177**	Maize (VitA)	Zambia	Vitamin A Maize	GV671A	2015	Vitamin A	11.4	
**178**	Maize (VitA)	Zambia	Vitamin A Maize	GV672A	2015	Vitamin A	10.2	
**179**	Maize (VitA)	Zambia	Vitamin A Maize	GV673A	2015	Vitamin A	10.9	
**180**	Maize (VitA)	Zambia	Vitamin A Maize	GV6023A	2019	Vitamin A	13	
**181**	Maize (VitA)	Zambia	Vitamin A Maize	GV6029A	2019	Vitamin A	14.9	
**182**	Maize (VitA)	Zambia	Vitamin A Maize	GV6027A	2019	Vitamin A	9.5	
**183**	Maize (VitA)	Zambia	Vitamin A Maize	GV6017A	2019	Vitamin A	10.8	
**184**	Maize (VitA)	Zambia	Vitamin A Maize	GV6025A	2019	Vitamin A	11.8	
**185**	Maize (VitA)	Zimbabwe	Vitamin A Maize	ZS242A	2015	Vitamin A	9.8	
**186**	Maize (VitA)	Zimbabwe	Vitamin A Maize	ZS244A	2016	Vitamin A	12	
**187**	Maize (VitA)	Zimbabwe	Vitamin A Maize	ZS246A	2016	Vitamin A	8.7	
**188**	Maize (VitA)	Zimbabwe	Vitamin A Maize	ZS248A	2016	Vitamin A	7.8	
**189**	Maize (VitA)	Zimbabwe	Vitamin A Maize	ZS500A	2019	Vitamin A	14.9	
**190**	Maize (Zn)	Colombia	Zinc Maize	BIO-MZn01	2018	Zinc	34.5	
**191**	Maize (Zn)	Colombia	Zinc Maize	SGBIOH6	2020	Zinc	32	
**192**	Maize (Zn)	Colombia	Zinc Maize	SGBIOH2	2019	Zinc	33	
**193**	Maize (Zn)	El Salvador	Zinc Maize	â€‹CENTA Porrillo 2020	2020	Zinc	TBD	
**194**	Maize (Zn)	Guatemala	Zinc Maize	ICTA HB-18ACP+Zn	2018	Zinc	31	

**195**	Maize (Zn)	Guatemala	Zinc Maize	ICTA B-15ACP+Zn	2018	Zinc	30
**196**	Maize (Zn)	Guatemala	Zinc Maize	Fortaleza 17	2020	Zinc	32
**197**	Maize (Zn)	Honduras	Zinc Maize	DICTA B02	2017	Zinc	34.5
**198**	Maize (Zn)	Honduras	Zinc Maize	DICTA B03	2017	Zinc	35.1
**199**	Maize (Zn)	Nicaragua	Zinc Maize	Fortinica	2018	Zinc	34.9
**200**	Maize (Zn)	Nicaragua	Zinc Maize	INTA-Nutremas	2018	Zinc	35
**201**	Pearl Millet	India	Iron Pearl Millet	Dhanashakti	2014	Iron	72
**202**	Pearl Millet	India	Iron Pearl Millet	Shakti 1201 (Dhoni)	2015	Iron	74
**203**	Pearl Millet	India	Iron Pearl Millet	HHB 299	2017	Iron	73
**204**	Pearl Millet	India	Iron Pearl Millet	AHB 1200 Fe	2017	Iron	77
**205**	Pearl Millet	India	Iron Pearl Millet	Phule Mahashakti (DHBH 1211)	2018	Iron	78
**206**	Pearl Millet	India	Iron Pearl Millet	HHB 311	2018	Iron	62
**207**	Pearl Millet	India	Iron Pearl Millet	RHB 234	2018	Iron	62
**208**	Pearl Millet	India	Iron Pearl Millet	AHB 1269 Fe	2018	Iron	73
**209**	Pearl Millet	India	Iron Pearl Millet	RHB 233	2018	Iron	64
**210**	Pearl Millet	India	Iron Pearl Millet	Moti Shakti (GHB 1225â€‹)	2019	Iron	TBD
**211**	Pearl Millet	Niger	Iron Pearl Millet	Chakti	2018	Iron	65
**212**	Rice	Bangladesh	Zinc Rice	BRRI Dhan62	2013	Zinc	20
**213**	Rice	Bangladesh	Zinc Rice	BRRI Dhan64	2014	Zinc	24
**214**	Rice	Bangladesh	Zinc Rice	BRRI Dhan72	2015	Zinc	23
**215**	Rice	Bangladesh	Zinc Rice	BRRI Dhan74	2015	Zinc	24.2
**216**	Rice	Bangladesh	Zinc Rice	BU Aromatic Hybrid Dhan-1	2016	Zinc	22
**217**	Rice	Bangladesh	Zinc Rice	Binadhan 20	2017	Zinc	27.5
**218**	Rice	Bangladesh	Zinc Rice	BU Aromatic Dhan-2	2016	Zinc	22
**219**	Rice	Bangladesh	Zinc Rice	BRRI Dhan84	2017	Zinc	27.6
**220**	Rice	Bolivia	Zinc Rice	CIAT BIO-44 +Zinc	2019	Zinc	22
**221**	Rice	Colombia	Zinc Rice	Fedearroz BIOZn 035	2020	Zinc	26
**222**	Rice	El Salvador	Zinc Rice	CENTA A-Nutremas	2019	Zinc	22.8
**223**	Rice	India	Zinc Rice	DRR Dhan 49	2018	Zinc	25.2
**224**	Rice	Indonesia	Zinc Rice	INPARI IR Nutri Zinc	2018	Zinc	25
**225**	Rice	Nicaragua	Zinc Rice	INTA Las Minas	2020	Zinc	TBD
**226**	Sorghum	India	Zinc Sorghum	Parbhani Shakti	2018	Zinc	32
**227**	Sweet Potato	Angola	Vitamin A Sweet Potato	Camuto	2013	Vitamin A	TBD
**228**	Sweet Potato	Angola	Vitamin A Sweet Potato	Cenoura	2013	Vitamin A	TBD
**229**	Sweet Potato	Angola	Vitamin A Sweet Potato	Helena	2013	Vitamin A	TBD
**230**	Sweet Potato	Angola	Vitamin A Sweet Potato	Zapallo	2013	Vitamin A	TBD
**231**	Sweet Potato	Angola	Vitamin A Sweet Potato	Nemanete	2013	Vitamin A	TBD
**232**	Sweet Potato	Angola	Vitamin A Sweet Potato	Huambachero	2013	Vitamin A	TBD
**233**	Sweet Potato	Angola	Vitamin A Sweet Potato	Mayai	2013	Vitamin A	TBD
**234**	Sweet Potato	Bangladesh	Vitamin A Sweet Potato	BARI-SP-12	2013	Vitamin A	TBD
**235**	Sweet Potato	Bangladesh	Vitamin A Sweet Potato	BARI-SP-13	2013	Vitamin A	TBD
**236**	Sweet Potato	Bangladesh	Vitamin A Sweet Potato	BARI-SP-14	2017	Vitamin A	TBD
**237**	Sweet Potato	Bangladesh	Vitamin A Sweet Potato	BARI-SP-15	2017	Vitamin A	TBD
**238**	Sweet Potato	Brazil	Vitamin A Sweet Potato	Beauregard	2010	Vitamin A	115
**239**	Sweet Potato	Brazil	Vitamin A Sweet Potato	BRS Amelia	2011	Vitamin A	TBD
**240**	Sweet Potato	Brazil	Vitamin A Sweet Potato	SCS-367 Favorita	2011	Vitamin A	TBD
**241**	Sweet Potato	Burkina Faso	Vitamin A Sweet Potato	Tiebele-2	2014	Vitamin A	TBD
**242**	Sweet Potato	Burkina Faso	Vitamin A Sweet Potato	Caromex	2005	Vitamin A	TBD
**243**	Sweet Potato	Burkina Faso	Vitamin A Sweet Potato	CIP199062.1	2005	Vitamin A	TBD
**244**	Sweet Potato	Burkina Faso	Vitamin A Sweet Potato	Bagre	2014	Vitamin A	TBD
**245**	Sweet Potato	Burkina Faso	Vitamin A Sweet Potato	Jewel	2014	Vitamin A	TBD
**246**	Sweet Potato	Burkina Faso	Vitamin A Sweet Potato	Nanyoumondo-1	2014	Vitamin A	TBD
**247**	Sweet Potato	Burkina Faso	Vitamin A Sweet Potato	Nanyoumondo-2	2014	Vitamin A	TBD
**248**	Sweet Potato	Burkina Faso	Vitamin A Sweet Potato	Heere	2019	Vitamin A	TBD
**249**	Sweet Potato	Burundi	Vitamin A Sweet Potato	Cacearpedo	2017	Vitamin A	TBD
**250**	Sweet Potato	Burundi	Vitamin A Sweet Potato	97062	2017	Vitamin A	TBD
**251**	Sweet Potato	China	Vitamin A Sweet Potato	Yanshu 25	2012	Vitamin A	36
**252**	Sweet Potato	China	Vitamin A Sweet Potato	Zheshu 13	2004	Vitamin A	TBD
**253**	Sweet Potato	China	Vitamin A Sweet Potato	Guangshu 87	2005	Vitamin A	TBD
**254**	Sweet Potato	China	Vitamin A Sweet Potato	Xiangxiang	2007	Vitamin A	TBD
**255**	Sweet Potato	Colombia	Vitamin A Sweet Potato	Agrosavia Aurora	2019	Vitamin A	214
**256**	Sweet Potato	East Timor	Vitamin A Sweet Potato	Hohrae-3	2007	Vitamin A	TBD
**257**	Sweet Potato	Ethiopia	Vitamin A Sweet Potato	Kulfo	2005	Vitamin A	210.6
**258**	Sweet Potato	Ethiopia	Vitamin A Sweet Potato	Tulla	2005	Vitamin A	146.1
**259**	Sweet Potato	Ethiopia	Vitamin A Sweet Potato	Kero	2005	Vitamin A	TBD
**260**	Sweet Potato	Ethiopia	Vitamin A Sweet Potato	Birtukane	2008	Vitamin A	TBD
**261**	Sweet Potato	Ethiopia	Vitamin A Sweet Potato	Ukr/Eju-10	2019	Vitamin A	124
**262**	Sweet Potato	Ethiopia	Vitamin A Sweet Potato	Ukr/Eju-13	2019	Vitamin A	95
**263**	Sweet Potato	Ghana	Vitamin A Sweet Potato	CRI-Bohye	2011	Vitamin A	243.1
**264**	Sweet Potato	Ghana	Vitamin A Sweet Potato	CRI-Apomuden	2005	Vitamin A	55
**265**	Sweet Potato	Ghana	Vitamin A Sweet Potato	SARI-Nan	2018	Vitamin A	383.7
**266**	Sweet Potato	Guatemala	Vitamin A Sweet Potato	ICTA Dorado-BC	2016	Vitamin A	133
**267**	Sweet Potato	Guatemala	Vitamin A Sweet Potato	ICTA Pacifico-BC	2016	Vitamin A	77
**268**	Sweet Potato	India	Vitamin A Sweet Potato	Sree Kanaka	2004	Vitamin A	100
**269**	Sweet Potato	India	Vitamin A Sweet Potato	Indira Madhur	2006	Vitamin A	35
**270**	Sweet Potato	India	Vitamin A Sweet Potato	Kamala Sundari	2008	Vitamin A	TBD
**271**	Sweet Potato	India	Vitamin A Sweet Potato	Bhu Sona	2017	Vitamin A	140
**272**	Sweet Potato	India	Vitamin A Sweet Potato	Bhu Kanti	2016	Vitamin A	TBD
**273**	Sweet Potato	India	Vitamin A Sweet Potato	Bhu Ja	2016	Vitamin A	TBD
**274**	Sweet Potato	Indonesia	Vitamin A Sweet Potato	Beta-1	2009	Vitamin A	TBD
**275**	Sweet Potato	Indonesia	Vitamin A Sweet Potato	Beta-2	2009	Vitamin A	TBD
**276**	Sweet Potato	Ivory Coast	Vitamin A Sweet Potato	Irene	2015	Vitamin A	TBD
**277**	Sweet Potato	Ivory Coast	Vitamin A Sweet Potato	Bela Bela	2015	Vitamin A	TBD
**278**	Sweet Potato	Ivory Coast	Vitamin A Sweet Potato	TIB-440060	2015	Vitamin A	TBD
**279**	Sweet Potato	Ivory Coast	Vitamin A Sweet Potato	TIB	2012	Vitamin A	TBD
**280**	Sweet Potato	Kenya	Vitamin A Sweet Potato	K-117	2009	Vitamin A	TBD
**281**	Sweet Potato	Kenya	Vitamin A Sweet Potato	Kenspot-3	2013	Vitamin A	14
**282**	Sweet Potato	Kenya	Vitamin A Sweet Potato	Kenspot-4	2013	Vitamin A	40
**283**	Sweet Potato	Kenya	Vitamin A Sweet Potato	Kenspot-5	2013	Vitamin A	55
**284**	Sweet Potato	Kenya	Vitamin A Sweet Potato	Vita	2013	Vitamin A	315
**285**	Sweet Potato	Kenya	Vitamin A Sweet Potato	K/KA2004/215	2015	Vitamin A	10
**286**	Sweet Potato	Kenya	Vitamin A Sweet Potato	Kabode	2013	Vitamin A	97
**287**	Sweet Potato	Kenya	Vitamin A Sweet Potato	To be confirmed	2019	Vitamin A	TBD
**288**	Sweet Potato	Kenya	Vitamin A Sweet Potato	Irene	2011	Vitamin A	333.2
**289**	Sweet Potato	Madagascar	Vitamin A Sweet Potato	Mendrika	2007	Vitamin A	TBD
**290**	Sweet Potato	Madagascar	Vitamin A Sweet Potato	BÃ´ra	2008	Vitamin A	46
**291**	Sweet Potato	Madagascar	Vitamin A Sweet Potato	Gloria	2011	Vitamin A	78
**292**	Sweet Potato	Madagascar	Vitamin A Sweet Potato	Zambezi	2011	Vitamin A	38

**293**	Sweet Potato	Madagascar	Vitamin A Sweet Potato	P162	2011	Vitamin A	TBD
**294**	Sweet Potato	Madagascar	Vitamin A Sweet Potato	J664	2015	Vitamin A	TBD
**295**	Sweet Potato	Madagascar	Vitamin A Sweet Potato	J314	2015	Vitamin A	TBD
**296**	Sweet Potato	Madagascar	Vitamin A Sweet Potato	Delvia	2017	Vitamin A	TBD
**297**	Sweet Potato	Madagascar	Vitamin A Sweet Potato	Jane	2017	Vitamin A	TBD
**298**	Sweet Potato	Madagascar	Vitamin A Sweet Potato	Lourdes	2017	Vitamin A	TBD
**299**	Sweet Potato	Madagascar	Vitamin A Sweet Potato	Irene	2017	Vitamin A	TBD
**300**	Sweet Potato	Madagascar	Vitamin A Sweet Potato	Erica	2017	Vitamin A	TBD
**301**	Sweet Potato	Malawi	Vitamin A Sweet Potato	Ana Akwanire	2011	Vitamin A	55
**302**	Sweet Potato	Malawi	Vitamin A Sweet Potato	Kadyaubwerere	2011	Vitamin A	89
**303**	Sweet Potato	Malawi	Vitamin A Sweet Potato	Kaphulira	2011	Vitamin A	32
**304**	Sweet Potato	Malawi	Vitamin A Sweet Potato	Mathuthu	2011	Vitamin A	29
**305**	Sweet Potato	Malawi	Vitamin A Sweet Potato	Zondeni	2008	Vitamin A	90
**306**	Sweet Potato	Malawi	Vitamin A Sweet Potato	Chipika	2011	Vitamin A	124.7
**307**	Sweet Potato	Mozambique	Vitamin A Sweet Potato	Ejumula	2011	Vitamin A	53.8
**308**	Sweet Potato	Mozambique	Vitamin A Sweet Potato	Persistente	2006	Vitamin A	TBD
**309**	Sweet Potato	Mozambique	Vitamin A Sweet Potato	Cordner	2006	Vitamin A	TBD
**310**	Sweet Potato	Mozambique	Vitamin A Sweet Potato	Amelia	2011	Vitamin A	50
**311**	Sweet Potato	Mozambique	Vitamin A Sweet Potato	Bela	2011	Vitamin A	83.9
**312**	Sweet Potato	Mozambique	Vitamin A Sweet Potato	Cecilia	2011	Vitamin A	60.1
**313**	Sweet Potato	Mozambique	Vitamin A Sweet Potato	Delvia	2011	Vitamin A	55.4
**314**	Sweet Potato	Mozambique	Vitamin A Sweet Potato	Erica	2011	Vitamin A	101.6
**315**	Sweet Potato	Mozambique	Vitamin A Sweet Potato	Esther	2011	Vitamin A	47.2
**316**	Sweet Potato	Mozambique	Vitamin A Sweet Potato	Ininda	2011	Vitamin A	53.1
**317**	Sweet Potato	Mozambique	Vitamin A Sweet Potato	Irene	2011	Vitamin A	60.6
**318**	Sweet Potato	Mozambique	Vitamin A Sweet Potato	Jane	2011	Vitamin A	55.9
**319**	Sweet Potato	Mozambique	Vitamin A Sweet Potato	Lourdes	2011	Vitamin A	99.4
**320**	Sweet Potato	Mozambique	Vitamin A Sweet Potato	Melinda	2011	Vitamin A	57.1
**321**	Sweet Potato	Mozambique	Vitamin A Sweet Potato	Namanga	2011	Vitamin A	83.9
**322**	Sweet Potato	Mozambique	Vitamin A Sweet Potato	Sumaia	2011	Vitamin A	77
**323**	Sweet Potato	Mozambique	Vitamin A Sweet Potato	Tio Joe	2011	Vitamin A	103.2
**324**	Sweet Potato	Mozambique	Vitamin A Sweet Potato	Gaba Gaba	2006	Vitamin A	TBD
**325**	Sweet Potato	Mozambique	Vitamin A Sweet Potato	Caelan	2016	Vitamin A	TBD
**326**	Sweet Potato	Mozambique	Vitamin A Sweet Potato	Ivone	2016	Vitamin A	70.6
**327**	Sweet Potato	Mozambique	Vitamin A Sweet Potato	Victoria	2016	Vitamin A	47.7
**328**	Sweet Potato	Mozambique	Vitamin A Sweet Potato	Alisha	2016	Vitamin A	73.3
**329**	Sweet Potato	Mozambique	Vitamin A Sweet Potato	Lawrence	2016	Vitamin A	62.5
**330**	Sweet Potato	Nicaragua	Vitamin A Sweet Potato	INTA Nutritivo	2017	Vitamin A	TBD
**331**	Sweet Potato	Nigeria	Vitamin A Sweet Potato	King J	2012	Vitamin A	TBD
**332**	Sweet Potato	Nigeria	Vitamin A Sweet Potato	Mothersâ€™ delight	2013	Vitamin A	TBD
**333**	Sweet Potato	Nigeria	Vitamin A Sweet Potato	Solo Gold	2018	Vitamin A	TBD
**334**	Sweet Potato	Panama	Vitamin A Sweet Potato	IDIAP C9017	2017	Vitamin A	52
**335**	Sweet Potato	Panama	Vitamin A Sweet Potato	IDIAP C0317	2017	Vitamin A	58
**336**	Sweet Potato	Peru	Vitamin A Sweet Potato	Mejorada	2005	Vitamin A	TBD
**337**	Sweet Potato	Peru	Vitamin A Sweet Potato	Arne	2010	Vitamin A	TBD
**338**	Sweet Potato	Peru	Vitamin A Sweet Potato	Benjamin	2010	Vitamin A	TBD
**339**	Sweet Potato	Peru	Vitamin A Sweet Potato	Abigail	2014	Vitamin A	TBD
**340**	Sweet Potato	Peru	Vitamin A Sweet Potato	Isabel	2014	Vitamin A	TBD
**341**	Sweet Potato	Peru	Vitamin A Sweet Potato	Sumy	2014	Vitamin A	TBD
**342**	Sweet Potato	Rwanda	Vitamin A Sweet Potato	Cacearpedo	2008	Vitamin A	TBD
**343**	Sweet Potato	Rwanda	Vitamin A Sweet Potato	Kakamega	2006	Vitamin A	TBD
**344**	Sweet Potato	Rwanda	Vitamin A Sweet Potato	Terimbere	2013	Vitamin A	TBD
**345**	Sweet Potato	Rwanda	Vitamin A Sweet Potato	Ndamirabana	2013	Vitamin A	TBD
**346**	Sweet Potato	Rwanda	Vitamin A Sweet Potato	Gihingamukungu	2004	Vitamin A	TBD
**347**	Sweet Potato	Rwanda	Vitamin A Sweet Potato	2000-040	2008	Vitamin A	TBD
**348**	Sweet Potato	South Africa	Vitamin A Sweet Potato	Impilo	2008	Vitamin A	51
**349**	Sweet Potato	South Africa	Vitamin A Sweet Potato	Khano	2007	Vitamin A	TBD
**350**	Sweet Potato	South Africa	Vitamin A Sweet Potato	Purple Sunset	2009	Vitamin A	TBD
**351**	Sweet Potato	South Africa	Vitamin A Sweet Potato	Bophelo	2011	Vitamin A	67
**352**	Sweet Potato	South Africa	Vitamin A Sweet Potato	Serolane	2007	Vitamin A	TBD
**353**	Sweet Potato	South Africa	Vitamin A Sweet Potato	Isondlo	2007	Vitamin A	TBD
**354**	Sweet Potato	South Korea	Vitamin A Sweet Potato	Haepymi	2004	Vitamin A	TBD
**355**	Sweet Potato	South Korea	Vitamin A Sweet Potato	Dahomi	2012	Vitamin A	TBD
**356**	Sweet Potato	Tanzania	Vitamin A Sweet Potato	Mayai	2016	Vitamin A	110
**357**	Sweet Potato	Tanzania	Vitamin A Sweet Potato	Ejumula	2013	Vitamin A	144
**358**	Sweet Potato	Tanzania	Vitamin A Sweet Potato	Kakamega	2014	Vitamin A	38
**359**	Sweet Potato	Tanzania	Vitamin A Sweet Potato	Kiegea	2010	Vitamin A	18
**360**	Sweet Potato	Tanzania	Vitamin A Sweet Potato	Kabode	2016	Vitamin A	97
**361**	Sweet Potato	Uganda	Vitamin A Sweet Potato	Ejumula	2004	Vitamin A	91
**362**	Sweet Potato	Uganda	Vitamin A Sweet Potato	Kakamega	2004	Vitamin A	41
**363**	Sweet Potato	Uganda	Vitamin A Sweet Potato	Kabode	2007	Vitamin A	97
**364**	Sweet Potato	Uganda	Vitamin A Sweet Potato	Gerald	2013	Vitamin A	72
**365**	Sweet Potato	Uganda	Vitamin A Sweet Potato	Joweria	2013	Vitamin A	95
**366**	Sweet Potato	Uganda	Vitamin A Sweet Potato	Vita	2007	Vitamin A	75
**367**	Sweet Potato	Uganda	Vitamin A Sweet Potato	NASPOT 7	2007	Vitamin A	108
**368**	Sweet Potato	Uganda	Vitamin A Sweet Potato	NASPOT 8	2007	Vitamin A	144
**369**	Sweet Potato	Zambia	Vitamin A Sweet Potato	Chiwoko	2014	Vitamin A	110
**370**	Sweet Potato	Zambia	Vitamin A Sweet Potato	Olympia	2014	Vitamin A	49
**371**	Sweet Potato	Zambia	Vitamin A Sweet Potato	Chumfwa	2014	Vitamin A	342.1
**372**	Sweet Potato	Zambia	Vitamin A Sweet Potato	Kokota	2014	Vitamin A	TBD
**373**	Wheat	Bangladesh	Zinc Wheat	BARI-Gom33	2017	Zinc	33
**374**	Wheat	Bolivia	Zinc Wheat	INIAF Okinawa	2019	Zinc	TBD
**375**	Wheat	Brazil	Zinc Wheat	BRS 331	2012	Zinc	37.3
**376**	Wheat	India	Zinc Wheat	BHU-3	2014	Zinc	30.5
**377**	Wheat	India	Zinc Wheat	Zn-Shakti	2014	Zinc	34.2
**378**	Wheat	India	Zinc Wheat	BHU-1	2013	Zinc	34.8
**379**	Wheat	India	Zinc Wheat	BHU-5	2013	Zinc	29.5
**380**	Wheat	India	Zinc Wheat	WB-02	2017	Zinc	31
**381**	Wheat	India	Zinc Wheat	HPBW01	2017	Zinc	31
**382**	Wheat	India	Zinc Wheat	BHU-25	2018	Zinc	TBD
**383**	Wheat	India	Zinc Wheat	BHU-31	2018	Zinc	39.5
**384**	Wheat	India	Zinc Wheat	HUW 711	2019	Zinc	TBD
**385**	Wheat	Mexico	Zinc Wheat	Nohely F2018	2018	Zinc	31
**386**	Wheat	Nepal	Zinc Wheat	Zinc Gahun-1	2020	Zinc	TBD
**387**	Wheat	Nepal	Zinc Wheat	Himgange	2020	Zinc	TBD
**388**	Wheat	Nepal	Zinc Wheat	Panchakoshi	2020	Zinc	TBD
**389**	Wheat	Nepal	Zinc Wheat	Zinc Gahun-2	2020	Zinc	TBD
**390**	Wheat	Nepal	Zinc Wheat	Zinc wheat-3	2020	Zinc	TBD

**391**	Wheat	Nepal	Zinc Wheat	Borlaug100	2020	Zinc	TBD
**392**	Wheat	Pakistan	Zinc Wheat	Zincol-2016	2016	Zinc	37
**393**	Wheat	Pakistan	Zinc Wheat	Akhbar-2019	2020	Zinc	37

TBD - to be determined and validated using precision phenotyping
